# Investigation on the Concentration, Sources, and Photochemical Roles of Volatile Phenols in the Atmosphere in the North China Plain

**DOI:** 10.3390/toxics13090744

**Published:** 2025-08-31

**Authors:** Ziyan Chen, Kaitao Chen, Min Cai, Xingru Li

**Affiliations:** 1 Analysis and Testing Center, Department of Chemistry, Capital Normal University, Beijing 100048, China; zy.chen27@foxmail.com; 2 Key Laboratory of Geographic Information Science of the Ministry of Education, School of Geographic Sciences, East China Normal University, Shanghai 200241, China; ckt0106@126.com; 3 School of Environment, Beijing Normal University, Beijing 100875, China; cm990427@126.com

**Keywords:** the North China Plain, volatile phenols, reactivity, source apportionment

## Abstract

Volatile phenols in the atmosphere are important precursors of ozone and secondary organic aerosols (SOAs). Despite their importance, the lack of effective observation and analysis methods has led to less attention paid to them, leading to gaps in our understanding of their behavior and effects on atmospheric chemistry. This study aimed to evaluate the concentration levels, sources, and environmental impacts of volatile phenols in ambient air, focusing on the urban area of Beijing and the suburban district of Heze in the North China Plain during winter. Samples were collected using an XAD-7 column and analyzed by high-performance liquid chromatography with ultraviolet detection (UPLC-UV). Results indicated that a higher concentration of 11 detected phenols was found in Beijing than that in Heze, with the average concentration of 23.60 ± 8.99 ppbv and 18.38 ± 2.34 ppbv. Phenol and cresol with strong photochemical activity were the predominant species, accounting for about 52% (Heze) and 66% (Beijing) of the total phenols, which indicates that more attention should be paid to volatile phenols in urban areas. Higher levels of L_OH_ in Beijing (36.86 s^−1^) and Heze (22.06 s^−1^) compared to other studies about PAMS and carbonyls indicated that these volatile phenols play an undeniable role in atmospheric oxidation reactions. Positive Matrix Factorization (PMF) identified major sources as pesticide usage (15.6%), organic chemicals (31.9%), and combustion or secondary conversion (52.5%). These findings underscore the multifaceted impact of phenols, influencing both gaseous pollutant concentrations and particulate matter formation, with potential implications for environmental and public health.

## 1. Introduction

Oxygen-containing volatile organic compounds (OVOCs) are a diverse group of organic compounds that contain oxygen and have high vapor pressures at ambient temperatures. OVOCs have been receiving increasing attention for their adverse effects on both human health and the environment [1,2,3] and are also precursors of atmospheric oxidants and secondary organic aerosols (SOAs) [4]. OVOCs are present in the atmosphere, mainly composed of carbonyls, such as aldehydes and ketones, phenols, and alcohols. Among the various OVOCs, volatile phenols are particularly important components. They participate in photochemical reactions that lead to the formation of ozone and SOA, leading to serious air pollution [5]. Therefore, studying the composition, concentration levels, and atmospheric activity of volatile phenols in the atmosphere is of great significance for effectively reducing atmospheric photochemical pollution and the formation of SOA.

In recent years, researchers have employed a range of techniques to investigate the sources, emissions, and atmospheric transformations of volatile phenols. These include the use of gas chromatography and mass spectrometry to identify and quantify the compounds in various environmental samples, as well as the application of isotopic analysis to trace their origins. Additionally, modeling studies have been conducted to predict the dispersion and transformation patterns of volatile phenols in the atmosphere.

The sources of volatile phenols mainly include biomass combustion [6,7,8] and motor vehicle exhaust emissions [9,10]. In addition to the above emissions, the secondary transformations of atmospheric pollutants can also contribute significantly to the production of volatilized phenols [11,12]. For instance, photochemical reactions in the atmosphere, involving ultraviolet radiation and various gaseous pollutants, can lead aromatic compounds like benzene and toluene to the formation of phenolic compounds [13,14]. In China, various anthropogenic activities such as industrial production, waste incineration and agricultural practices are also major sources of volatile phenol emissions [15,16].

Phenolic compounds can undergo homogeneous or heterogeneous chemical transformations in the atmosphere, forming highly oxidizing and toxic SOA. Gas-phase reactions involving volatile phenols and these radicals are presumed to constitute the primary degradation mechanism in the atmosphere, owing to the highly reactive chemical properties of volatile phenols [17]. Specifically, during the day, the reaction of hydroxyl radicals (OH) with phenols primarily occurs through two pathways: first, OH addition to the aromatic ring, and second, hydrogen abstraction from the phenolic hydroxyl group or the aromatic ring. The phenoxy/aroxy radicals generated by these two pathways undergo further oxidation, producing hydroxylated products (such as catechol) and low-volatility products formed by ring opening after further oxidation. These low-volatility products tend to partition into the particulate phase and contribute to SOA. Under high NOx conditions, the OH pathway may also generate small amounts of nitro-substituted products via RO_2_/NO_x_ chemical reactions [18]. At night, nitrate radicals (NO_3_·) react rapidly with many phenols, often producing high-yield nitroaromatic products (nitrophenols, nitrocatechol, etc.). These nitro products are low in volatility and highly absorbent, making them key components of atmospheric ‘brown carbon’ and nighttime SOA formation [19,20]. Additionally, many phenols have high water solubility, and aqueous-phase chemistry (e.g., solution-phase OH, triplet-state organic-induced reactions, nitrite/nitrate-related reactions) can produce nitration, condensation, and oligomerization products, significantly increase SOA yield, and generate colored nitro/condensed products. Aqueous pathways often yield different product spectra and higher particle yields compared to gas-phase pathways, particularly in humid or foggy environments [21,22,23,24,25].

These compounds can have different optical properties and hygroscopic behavior compared to the original particles, affecting their role in radiative forcing and cloud formation. The key to understanding these secondary conversion processes and products is to understand the composition and concentration levels of phenolic precursors.

Although volatilized phenols occupy a crucial position in atmospheric chemistry, they have garnered inadequate attention, primarily attributed to the exclusion of phenolic compounds from current analytical methodologies employed for Photochemical Assessment Monitoring Stations. Regrettably, this oversight has led to a substantial void in our comprehension of the environmental consequences of volatilized phenols. While significant progress has been made in understanding the chemistry of other PAMS, the phenolic subset remains largely uncharted territory. This is particularly concerning given the potential influence of these compounds on air quality and climate change. To bridge this knowledge gap, it is imperative to devise and adopt more sensitive and specific analytical techniques for the detection and quantification of volatilized phenols. Such techniques would not only enrich our understanding of their atmospheric behavior but also assist in devising effective strategies to mitigate their potential environmental impacts. By affording these phenolic compounds the due attention, we can acquire a more comprehensive understanding of their role in atmospheric chemistry and the broader implications for our environment.

Some research has been conducted on the importance of volatile phenols, and a series of studies have been conducted on their analysis methods and activity [26,27,28,29]. Various analytical techniques, such as spectrophotometry [30,31], gas chromatography [32], and gas chromatography–mass spectrometry [26,33], have been employed to detect phenols. The monitoring and analysis of atmospheric phenolic compounds have become standardized procedures, and comprehensive analytical methodologies have been devised to cater to this requirement. For example, a method for the determination of phenol, cresol isomers, catechol, and guaiacol in atmospheric particulate matter (PM) using liquid chromatography–(ESI)–mass spectrometry (LC-(ESI)-MS) has been described [21]. This approach incorporated a derivatization pretreatment process using DnsCl and successfully analyzed phenolic compounds present in seasonal PM samples collected from Ljubljana, Slovenia. And Chen et. successfully established an ultra-high-performance liquid chromatography (UPLC) method for the analysis of phenolic compounds present in the atmosphere. This method efficiently separated and accurately quantified 11 phenolic compounds at a wavelength of 280 nm [34].

Despite the advances in analytical techniques for the detection of phenolic compounds, it is worth noting that reliable observational data on volatile phenolic chemicals in China’s atmosphere are still scarce and mostly confined to a single region (e.g., Beijing [35], Yangtze River Delta [36], etc.). Data on the North China Plain, especially the less developed regions of the country, are still scarce. Therefore, further research and monitoring efforts are imperative to deepen our understanding of these compounds and their presence in the atmosphere.

To address the issue of atmospheric emissions of volatile phenolic compounds in two distinct regions within the North China Plain, namely, the urban locale of Beijing and the rural locale of Heze in Shandong province, we have developed a solid-phase solvent extraction–high-performance liquid chromatography (SPE-UPLC) technique. This method exhibits stable chromatographic peaks, offers a shortened detection time, and demonstrates high analytical sensitivity. Additionally, we calculated the hydroxyl radical depletion rate (L_OH_) and ozone generation potential (OFP) of phenols to evaluate their atmospheric chemical activity and to investigate their contribution to atmospheric pollution, especially ozone pollution. We hope to provide new perspectives on the management of atmospheric ozone. Concurrently, we employed the Positive Matrix Factorization (PMF) model to investigate the sources of volatile phenols during the winter season in the central Beijing area. This comprehensive approach aims to provide crucial technical support for the implementation of effective air pollution control strategies and proactive emission reduction measures.

## 2. Materials and Methods

### 2.1. Sample Collection

**Figure 1:**
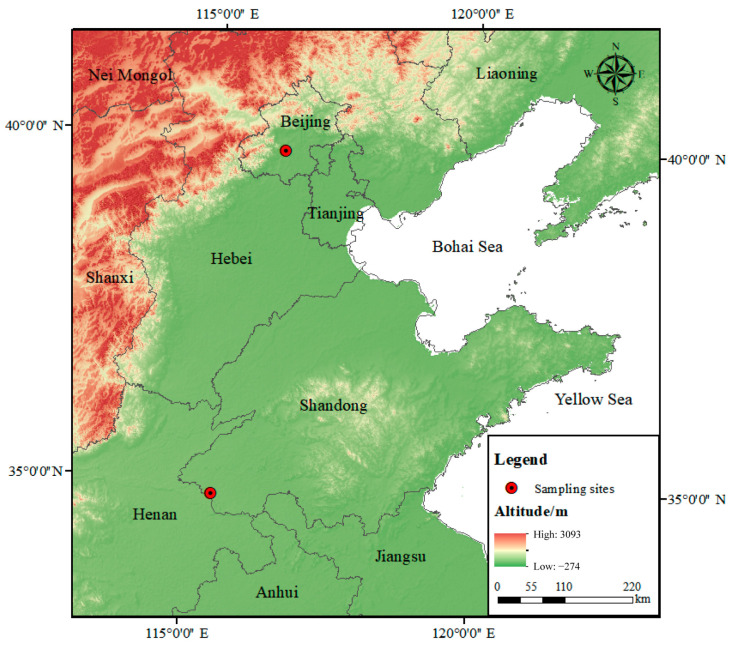
Map of sampling sites in the North China Plain.

Two sampling sites for volatile phenols were set up in Beijing (N 39°56′10.36″, E 116°18′43.44″) and Heze (N 34°51′1.23″, E 115°24′28.63″) in the North China Plain. In December 2022 and March 2023, samples were taken at the two sampling sites at the same time. The sampling sites are shown in Figure 1. The Beijing station is located in the center of the city, with convenient transportation around it. It is adjacent to the transportation artery and relies on the West Third Ring Road, the main road of Beijing. There are gas stations, restaurants, residential areas, etc. nearby, which have typical urban characteristics. The sampling site in Heze is located in a village, which is ecological wetlands along the Yellow River Road near the site, close to the crop planting land; local residents are mainly engaged in agricultural activities, the cooking method is mainly wood burning and natural gas, and there are no enterprises with serious pollution emission such as factories in the vicinity. The sampling procedures followed the “Technical guidelines for fugitive emissions monitoring of air pollutants” (HJ/T55-2000). Air samples were collected using an air sampler (QC-IB, Beijing Ke’an Labor Insurance New Technology Co., Ltd., Beijing, China) and XAD-7 resin sampling tubes with an inner diameter of 6 mm, an outer diameter of 8 mm, and a length of 11 mm. The sample flow rate was set at 0.3 L/min, and the sampling duration was 4 h. Sampling periods were divided into three time slots: 8:00–12:00 in the morning, 12:00–16:00 in the afternoon, and 16:00–20:00 in the evening. The collected samples were stored below 4 °C in the dark, and analyses were completed in a timely manner. Beijing obtained a total of 57 valid samples, including 6 parallel double samples (accounting for 10.5%, meeting the standard requirements), and 4 blank samples (2 field blanks, 2 laboratory blanks). Similarly, Heze obtained a total of 60 valid samples, including 6 parallel double samples (accounting for 10.0% of the total and fulfilling the standard requirements), along with 7 blank samples (consisting of 5 field blanks and 2 laboratory blanks).

### 2.2. Sample Analysis

The analytical procedure referred to the standard “Ambient air—Determination of phenolic compounds by high-performance liquid chromatography” (HJ638-2012). Prior to any analysis, the sampling tubes were eluted by a slow addition of 5 mL methanol (HPLC Grade, 99.9%, Fisher Chemical, Waltham, MA, USA) using a solid-phase extraction device for elution. The eluant was collected using a 5 mL centrifuge tube and gently evaporated to just below the 2 mL mark using a gentle stream of nitrogen gas (OA-SISTM Hea ting System, N-EVAPTM 116, Berlin, MA, USA). The resulting solution was then filtered through an organic phase filter membrane with a pore size of 0.22 μm, and then analyzed using a high-performance liquid chromatography system equipped with an ultraviolet detector (UPLC-UV) (model UltiMate 3000, manufactured by Thermo Fisher, Waltham, MA, USA).

A column of Acclaim 120 C18 (5 μm, 25 cm × 4.6 mm) was used. The injection volume was 10.0 μL, the detection wavelength was 280 nm, the flow rate was 1.5 mL/min, and the mobile phase was acetonitrile (HPLC Grade. 99.95%, Fisher Chemical, Waltham, MA, USA) and ultrapure water (Cascada^TM^, Pall, Port Washington, NY, USA) containing 0.1% formic acid (HPLC Grade, 99%, DIKMA, Shanghai, China). The analytical method was 30% acetonitrile/70% water held for 7.5 min, then a linear change to 55% acetonitrile/45% water held for 2 min, then a change to 80% acetonitrile/20% water held for 1.5 min before changing to 100% acetonitrile/0% water, with an additional 5 min equilibrium method of linearly changing 100% acetonitrile/0% water to 30% acetonitrile/70% water before running the next sample. The analysis involved identifying the retention time of the standard samples and quantifying them using the external standard method.

According to Supplementary Materials Table S1, comprehensive details regarding volatile phenols are provided, encompassing their molecular formulas, structural configurations, correlation coefficients, method detection limits (MDLs), precision, and spiked recoveries. Specifically, the MDLs range from 0.15 to 0.69 ppbv. Additionally, relative standard deviations (RSDs) were calculated for each compound, revealing values less than 8.0% for eight measurements of medium-concentration (0.5 mg/L) standard samples. This underscores the high precision of the instrumentation utilized in the current study. Furthermore, the assessment of accuracy through the determination of spiked recoveries, ranging from 96.6% to 126.2% with acceptable errors, serves as a crucial validation step.

A strict pre- and post-acquisition flow variation of less than 5% was used for all analytical processes, which were carried out under strict quality assurance and quality control. We ensured that at least 1 blank sample per batch was determined and the results were below the MDLs, as well as at least 10% of parallel samples were determined for per batch of samples.

### 2.3. Evaluation of Ozone and Secondary Organic Aerosol Precursors

#### 2.3.1. Rate of Volatile Phenolic Hydroxyl Group Depletion

The presence of phenolic groups in volatile phenolic compounds results in high reactivity, particularly during photochemical oxidation. These compounds readily react with atmospheric oxidants, such as the hydroxyl radical (OH), to produce secondary pollutants, including ozone and secondary organic aerosols (SOAs). Consequently, they represent a crucial component of atmospheric chemistry, often exerting a significant impact on the atmospheric oxidizing capacity. The hydroxyl radical depletion rate calculation method, as proposed by Roger Atkinson [37], was employed to assess the photochemical reactivity of volatile phenols.

The OH radical loss rate is calculated using Equation (1):

(1) $$L_{OHi}=K_{OHi}\times \left[{phenols}_{i}\right]$$

in which L_OHi_ is the OH radical loss rate of the ith VOC species in s^−1^; and K_OHi_ (cm^3^·mol^−1^·s^−1^) is the rate constant of reactions between the ith phenols species and OH radicals. The ·OH reaction rate coefficients (K_OH_) at 298 K are derived from the work of Atkinson Roger [38], and [phenol_i_] is the measured concentration of the ith species in μg/m^3^. Although the temperature during the winter sampling period of this study was below 298 K, the ·OH reaction rate constant (K_OH_) used was still based on values obtained at 298 K. This approach is widely adopted in existing studies, primarily because most phenolic compounds lack complete Arrhenius parameters, and the K_OH_ of aromatic compounds exhibits relatively weak temperature dependence within the common temperature range of the troposphere. To validate the robustness of the results, we conducted a sensitivity analysis in the Supplementary Materials, varying the K_OH_ within a ±20% range. The results showed that changes in L_OH_ were all within 20%, indicating that using the K_OH_ value at 298 K introduces only limited uncertainty and does not affect the main conclusions of this paper. Detailed explanations and sensitivity results based on ±20% K_OH_ are provided in Supplementary Materials Table S2 and Text S2 [39,40].

#### 2.3.2. Ozone Formation Potential of VOCs

In contrast to hydroxyl depletion rates, which primarily focus on the immediate reaction kinetics of compounds, ozone formation potential (OFP) is employed to evaluate the contribution of compounds to ground-level ozone concentrations over extended periods. This assessment is of significant importance for understanding the formation of photochemical smog and for the development of effective air quality management strategies.

The calculation of OFP is based on the maximum incremental reactivity (MIR) methodology developed by Carter [41]. MIR quantifies the maximum reactivity of individual VOC species under high NOx conditions, where ozone formation is most sensitive to VOCs concentrations. The OFP for each phenol species is determined by multiplying the individual VOC concentration by its respective MIR value, as shown in Equation (2):

(2) $${OFP}_{i}={MIR}_{i}\times \left[{phenol}_{i}\right]$$

where OFP_i_ is the ozone formation potential of the ith VOC species (ppbv); and MIR_i_ is the MIR scale of the ith VOC species. The MIR coefficients for VOCs species are obtained from Carter [41]. [VOC_i_] is the measured concentration of the ith species (μg/m^3^). Similarly, the MIR values and reasons are provided in Supplementary Materials Table S2 and Text S3, respectively.

## 3. Results and Discussion

### 3.1. Molecular Composition

**Figure 2:**
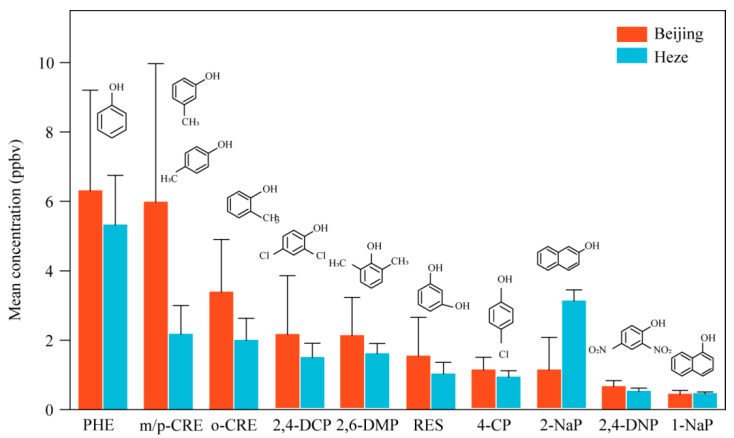
Mean concentrations of 11 phenolic compounds of Beijing and Heze during winter period. RES: resorcinol; PHE: phenol; o-CRE: o-cresol; *m/p*-CRE: *m-*, *p*-cresol; 2,4-DNP: 2,4-dinitrophenol; 4-CP: 4-chlorophenol; 1-NaP: 1-naphthol; 2-NaP: 2-naphthol; 2,6-DMP: 2,6-dimethylphenol; 2,4-DCP: 2,4-dichlorophenol. The top graphics of the bar are the structure of the volatile phenols.

In the present research, 11 phenolic compounds in the atmosphere, which included resorcinol (RES), phenol (PHE), o-cresol (o-CRE), *m/p*-cresol *(m/p*-CRE), 2,4-dinitrophenol (2,4-DNP), 4-chlorophenol (4-CP), 1-naphthol (1-NaP), 2-naphthol (2-NaP), 2,6-dimethylphenol (2,6-DMP), and 2,4-dichlorophenol (2,4-DCP), were analyzed. The mean total concentration during winter in Beijing and Heze were 23.60 ± 8.99 ppbv and 18.38 ± 2.34 ppbv, ranging from 19.60 to 35.50 ppbv and 16.86–19.93 ppbv, respectively. Among the detected 11 phenols, PHE had the highest concentration, an observed 6.30 ppbv in Beijing and 5.32 ppbv in Heze (Figure 2). Despite the limited availability of phenol-related reports, several studies have documented the concentrations of volatile phenols. The concentrations observed in our current study are significantly elevated compared to those reported in prior investigations. Specifically, the values reported for Jinan are 16.7 ± 3.7 ng/m^3^ [9], while the concentrations range from 6.5 to 10.4 ng/m^3^ for Strasbourg [26] and 114 ng/m^3^ for Santiago [42]. This variance can primarily be attributed to differences in the sampling methodologies employed. It is noteworthy that prior studies primarily utilized PUF membranes for capturing and enriching atmospheric phenols. However, given the high flow rates utilized in these studies, the enrichment efficiency may have been significantly lower compared to our current study.

Our study maintained a sampling flow rate of 300 mL/min, achieving an enrichment efficiency ranging from 50% to 65%. A slight decrease in enrichment efficiency was observed when the sampling flow rate increased to 400 mL/min (Supplementary Materials Figure S1). In addition, the method for the detection of volatile phenolic compounds using UPLC in this study did not involve high-temperature treatment steps such as derivatization during pretreatment, further avoiding sample loss. Consequently, it is plausible that prior studies may have underestimated the actual phenol concentrations in the atmosphere. Therefore, it is imperative to recognize that the actual concentration of volatile phenols in the atmosphere is higher than previously reported. This necessitates increased attention to the potential photochemical and fine-particle pollution that these compounds may cause.

Additionally, *m/p*-CRE and o-CRE had higher concentrations in Beijing (5.98 ppbv and 3.39 ppbv, respectively) than that in Heze (2.17 ppbv and 1.99 ppbv, respectively). Research indicates that phenol and cresol isomers, along with benzene-diphenol isomers, are prevalent in smoke from biomass combustion, accounting for approximately 14% to 25% of the mass of organic compounds in both gas and particulate phases [7]. Given that Beijing has more developed transportation and more congested vehicles compared to Heze, it is likely that motor vehicle exhaust [9] contributes significantly to the higher concentrations observed in Beijing. Furthermore, 2-NaP concentrations in Heze were notably higher (3.07 ppbv) than in downtown Beijing (1.08 ppbv). This is linked to various industrial processes, including those in the chemical, paper, paint, and pesticide sectors, where 2-NaP is produced as a byproduct [43]. Pesticide degradation also contributes to emissions of 2-NaP, making it a significant contaminant source in different regions.

In summary, phenols were generally more prevalent in the Beijing metropolitan area than in the Heze countryside throughout the winter period, with the exception of 2-NaP (Beijing had less than Heze). PHE, *m/p*-CRE, and o-CRE dominated the volatile phenols fractions in Beijing, while PHE and 2-NaP prevailed in Heze.

### 3.2. Characterization of Daily Changes in Phenols Under Different Pollution Conditions

**Figure 3:**
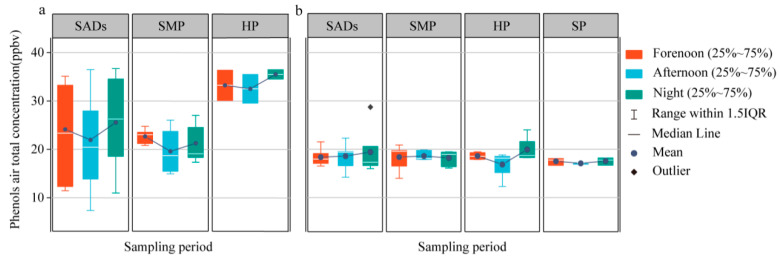
Daily change characteristics of phenols under different pollution conditions in winter of Beijing (**a**) and Heze (**b**). SADs: superior air quality days, SMP: slight-moderate pollution, HP: heavy pollution, SP: severe pollution. Air quality is determined based on the AQI index of the day—SAD: 0–100, SMP: 101–200, HP: 201–300HP, SP > 300. The AQI index is obtained from the website of China Environmental Monitoring General Station: https://www.cnemc.cn/ (accessed on 27 April 2025).

Figure 3 illustrates the diurnal variations in the total concentration of 11 phenols across various pollution levels in Beijing and Heze. In Beijing, the overall concentration of these 11 phenols exhibited a consistent trend across all pollution levels, initially decreasing and subsequently increasing (Figure 3a). This trend was characterized by the presence of a morning peak between 8:00 and 12:00, followed by an evening peak between 16:00 and 20:00. This pattern may be due to the fact that as the light intensity increases in the afternoon, the photochemical reaction of phenols also increases, leading to a decrease in phenol concentration during this period. This is because photolysis and hydroxyl radical oxidation become more active under intense light radiation. Furthermore, the morning and evening peaks in phenol concentrations were likely influenced by increased emissions from automotive exhausts, particularly those of PHE and cresol isomers (Supplementary Materials Figure S2).

Volatile phenols are typically associated with industrial and combustion emissions [44]. It can be inferred that volatile phenols are primarily influenced by anthropogenic activities, leading to higher concentrations in areas with substantial human activity. Furthermore, the sampling locations in Heze were situated in rural areas with relatively limited human activity compared to economically thriving urban areas like Beijing. In Heze, the impact of biomass combustion is notably more significant. Local cooking practices in Heze often involve the use of burning firewood, such as corn cobs and popular twigs, which are greatly affected by the smoke emitted during biomass combustion. These sources produce phenolic compounds as well, but with more diffuse temporal patterns and less intensity than traffic-related emissions. This circumstance helps elucidate why the daily trend in phenols in Heze were not as pronounced as those observed in Beijing. Furthermore, in the troposphere, biomass burning results in an elevation of O_3_ concentration. During the sampling period, the ozone atmospheric concentration at the Heze site was 52.76 ± 24.93 μg/m^3^, which was much larger than that in Beijing (36.56 ± 19.76 μg/m^−3^). This, in turn, intensifies the oxidative capacity for phenols depletion, further reducing the atmospheric concentration of phenols.

When contextualized within a broader regional framework, the observed differences between Beijing and Heze underscore how socio-economic development levels and energy usage patterns shape local air pollutant profiles. Similar results have been observed in other Chinese cities, such as Guangzhou and Chengdu [45,46,47], where urban areas exhibit higher phenol levels, driven by transportation and industrial emissions. This suggests that strategies for phenol pollution control should be tailored to local emission characteristics. For instance, urban areas might benefit from stricter vehicle emission controls, whereas rural areas like Heze could prioritize cleaner domestic energy alternatives to reduce biomass burning emissions.

In summary, the frequency of human activities has a distinct impact on the atmospheric concentration of volatile phenols. These compounds tend to exhibit higher concentrations in areas with elevated human activity. Moreover, there is a consistent daily trend, with concentrations typically peaking in the forenoon and evening and declining in the afternoon, regardless of the prevailing pollution levels.

### 3.3. Correlation Analysis Between Phenolic Compounds

**Figure 4:**
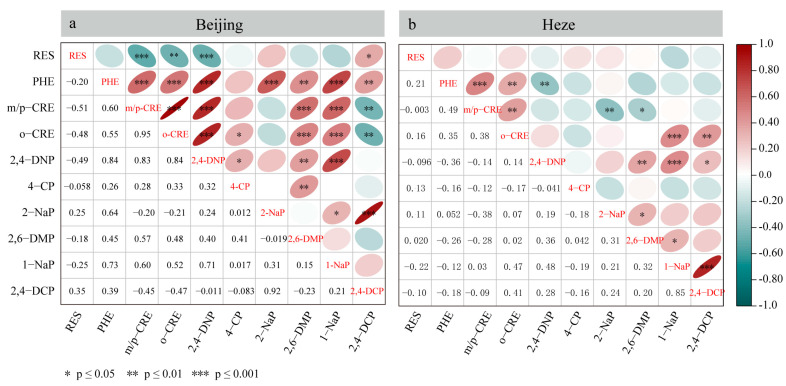
Heat map of correlation between phenolic compounds of Beijing (**a**) and Heze (**b**).

To elucidate the similarities in sources or formation pathways of phenolic compounds, Pearson correlation analyses were performed on winter observations from Beijing (Figure 4a) and Heze (Figure 4b). The results reveal distinct correlation patterns that reflect region-specific emission characteristics and atmospheric processes.

In Beijing (Figure 4a), significant correlations among 11 phenols highlight complex source interactions. RES exhibits moderate negative correlations with *m/p*-CRE (−0.51), o-CRE (−0.48), and 2,4-DNP (−0.49), suggesting divergent origins between this industrial/combustion tracer and phenolic compounds associated with secondary atmospheric reactions [48]. A distinct cluster comprising PHE, *m/p*-CRE, o-CRE, 2,4-DNP, 2,6-DMP, and 1-NaP shows strong positive correlations (r = 0.55–0.84), indicative of shared sources dominated by biomass combustion, vehicle emissions, and photochemical formation.

The compound 4-CP exhibits dual source characteristics: weak positive correlations with RES (0.28) and PHE (0.26) suggest partial source overlap, while its moderate association with 2-NaP (0.64) and negative correlations with *m/p*-CRE (−0.20) and o-CRE (−0.20) reveal predominant linkages to chemical wastewater emissions rather than combustion processes. This dichotomy underscores the compound’s multiple origin pathways.

In Heze (Figure 4b), weakened positive correlations among PHE, m/*p*-CRE, and o-CRE (0.35–0.49) suggest reduced source homogeneity compared to Beijing [49]. Emerging agricultural/industrial influences are evidenced by o-CRE’s moderate correlations with 1-NaP (0.38) and 4-CP (0.41), along with the strong 4-CP-1-NaP correlation (0.64) [50]. Negative correlations between PHE and 2,4-DNP (−0.36) and *m/p*-CRE-2-NaP (−0.38) highlight unique atmospheric partitioning mechanisms, potentially driven by enhanced humidity (RH > 80% during observations) promoting wet deposition of nitrophenols and methylated phenols [49].

### 3.4. Reactivity of Phenolic Compounds

The chemical reactivity of volatile phenols in the atmosphere represents a crucial factor in evaluating their impact on the environment and human health. These compounds are capable of reacting with atmospheric oxidants, including hydroxyl radicals and ozone, and participating in the atmospheric oxidation process, which affects the chemical composition of the atmosphere and environmental quality. In this study, the hydroxyl degradation rate (OH reactivity, L_OH_) and the maximum incremental OFP of volatile phenols were calculated in order to evaluate their activity in atmospheric chemical processes.

#### 3.4.1. Daily Time Series Characteristics

Based on the observations from this study and publicly available data from the China Environmental Monitoring General Station: https://www.cnemc.cn (accessed on 27 April 2025), Figure 5 shows the daily average concentrations of phenolic compounds and the daily average trends of meteorological/pollution parameters during the observation period of several dozen days in the study area. It is important to note that daily averaging smooths out the pronounced diurnal peaks and troughs within a day, so the amplitude observed in the figure better reflects the combined effects of “daily-scale net emission intensity, regional transport, and daytime cumulative photochemical conversion,” rather than instantaneous commuting or emission peaks.

**Figure 5:**
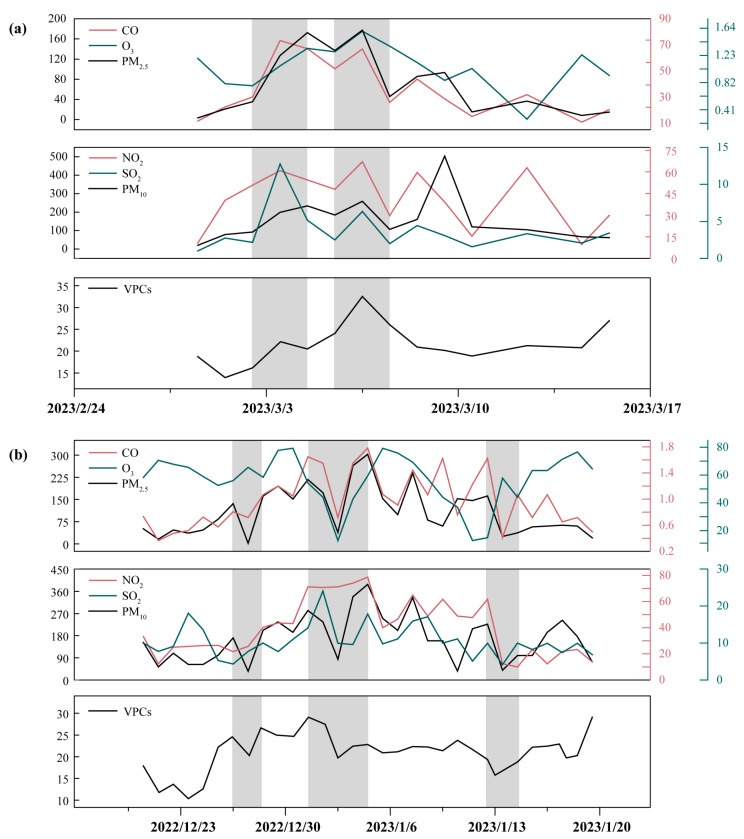
Trends in various parameters and volatile phenolic compounds (VPCs) at the Beijing (**a**) and Heze (**b**) stations. The shaded areas indicate typical periods of high ozone/heavy pollution. The units for each parameter are as follows: CO (mg/m^3^); O_3_, PM_2.5,_ NO_2_, SO_2_, PM_10_ (μg/m^3^); VPCs (ppbv).

At the Beijing station (Figure 5a), we observed several instances where daily average phenolic concentrations increased in tandem with CO and NO_x_, accompanied by enhanced PM_2.5_ levels, suggesting that these days were dominated by either single-source strong emissions (e.g., transportation, industry, or solid fuel combustion) or regional accumulation. If subsequent days show rising O_3_ levels and declining phenolic concentrations, this indicates that under stronger photochemical conditions, phenolics are consumed or converted into secondary products (e.g., nitration products or SOA precursors) as reactive precursors. From a chemical mechanism perspective, phenols are typically rapidly oxidized by OH during the day to form polar/low-volatility products; at night, under high NO_x_/NO_3_ conditions, nitration can occur to form nitrophenols and other products. Both pathways have been reported in the literature and can coexist during the same observation period [51].

In contrast, Heze Station (Figure 5b) exhibits a relatively flat daily average time series, with phenolic compounds maintaining high daily average values on some observation days, and sometimes rising in phase with or lagging behind O_3_. This behavior is more likely to reflect the daily-scale cumulative effects caused by regional secondary formation or long-range transport: after material accumulation due to prolonged transport times or boundary layer evolution (such as low mixing layers at night), O_3_ and secondary products derived from precursors may simultaneously increase under photochemical activation during the following daytime, thereby forming a coupled increase in phenolic compounds and O_3_ at the daily average level. Yuan et al. and others noted in field observations that the nitration pathway can produce significant accumulation of nitrophenols at night, thereby altering the daily concentration pattern [52].

Several days with high PM_2.5_ concentrations in the figure were accompanied by elevated levels of gaseous phenols, suggesting two non-mutually exclusive explanatory pathways: first, a common primary source (e.g., combustion emissions) simultaneously produces gaseous phenols and particulate matter precursors; second, gas-phase phenols undergo photochemical oxidation/nitration to produce low-volatility products that migrate to the particulate phase (becoming SOA precursors), resulting in a coupled increase in gas-phase phenols and PM_2.5_ at the daily average scale. These mechanisms are supported by field observations and box/0-D simulation studies, indicating that static daily average OFP/L_OH_ indicators alone are insufficient to fully characterize the time-resolved role of phenols in O_3_ and SOA formation [51,52].

More importantly, the background chemical state (sensitivity to O_3_-NO_x_-VOC) significantly modulates the “marginal effect” of phenols. According to Sillman et al.’s indicator quantity/ratio framework (e.g., VOC/NO_x_ or HCHO/NO_2_ indicators), when a region is in a VOC-limited state (high NO_x_, relatively low VOC), the marginal contribution of a single VOC category (even if it has high photochemical reactivity per unit mass) to O_3_ is often suppressed. Conversely, in NO_x_-limited conditions or when the VOC pool lacks highly reactive precursors, this contribution is amplified. Therefore, although phenols often exhibit high L_OH_/MIR (high unit mass reactivity), their actual impact on regional O_3_ must be interpreted within the framework of NO_x_ background and overall VOC pool activity. Based on the site differences shown in Figure 5 (Beijing: more periods with high NO_x_ levels; Heze: stronger local or regional transport influences), we infer that the marginal impact of phenols is suppressed on certain high NO_x_ days in Beijing, while their photochemical contribution is relatively enhanced during certain periods in Heze. To draw quantitative conclusions, further sensitivity simulations or observational constraint analyses are needed to obtain numerical estimates of RIR or P (O_3_).

#### 3.4.2. OH Loss Rate (L_OH_)

**Figure 6:**
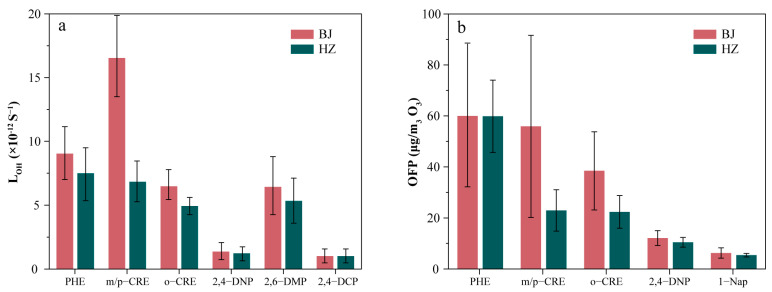
Photochemical reactivity of volatile phenolic compounds in Beijing and Heze. (**a**) is the hydroxyl radical depletion rate; (**b**) is the ozone generation potential.

The L_OH_ results for volatile phenolic compounds in the two regions are shown in Figure 6a. The L_OH_ values of volatile phenols in Beijing and Heze were calculated to be 36.86 × 10^−12^ s^−1^ and 22.06 × 10^−12^ s^−1^, respectively. Previous reports indicate that the L_OH_ of non-methane hydrocarbon-like PAMS in Beijing was reported to be 11 × 10^−12^ s^−1^ [35,53]. In cities with similar urban structures, such as Xianyang and Tongchuan in Shanxi province, the total L_OH_ of OVOCs (aldehydes and ketones) and NMHCs was reported to be 20.2 × 10^−12^ s^−1^ and 17.7 × 10^−12^ s^−1^, respectively [54]. In Xiamen SAR, a highly developed city with a similar economic level to Beijing, the L_OH_ of VOCs was reported to be 18.6 × 10^−12^ s^−1^ [55]. The L_OH_ of VOCs in cities with a comparable urban structure to Heze, such as Baoding and Cangzhou in Hebei province, has been reported to be 13.02 × 10^−12^ s^−1^ and 14.72 × 10^−12^ s^−1^, respectively [56]. In Baoji, a highly developed city with an economic level comparable to Heze, the L_OH_ of VOCs was reported to be 10.3 × 10^−12^ s^−1^ [57]. For detailed comparison results, please refer to Supplementary Materials Table S3.

As anticipated, the results indicated that the L_OH_ values of volatile phenolic compounds were significantly higher than those of PAMS and carbonyls. This finding is consistent with previous studies conducted in other regions. Prior research has indicated that volatile phenolic compounds rapidly generate secondary organic aerosols and other oxidized products in the atmosphere through reactions with hydroxyl radicals, thereby affecting air quality [20,29,58]. This study further demonstrates that volatile phenols exhibit higher reactivity in atmospheric chemical reactions. In contrast, PAMS have lower reactivity, and while they also generate ozone and organic aerosols, their contribution is relatively minor. This leads to the conclusion that volatile phenolic compounds play a more significant role in atmospheric oxidation.

Existing air quality monitoring and pollution control strategies primarily focus on common PAMS and carbonyls. However, the results of this study suggest that volatile phenolic compounds may have a more prominent impact on the atmospheric environment in certain cases due to their unique chemical structures and atmospheric chemical reaction mechanisms. It is recommended that volatile phenolic compounds with high L_OH_ values receive greater attention in air quality management and pollution control. Consequently, policymakers and environmental scientists should consider incorporating volatile phenolic compounds into routine monitoring and control for a more comprehensive air quality assessment and improvement.

In Beijing, the dominant substance reacting with ·OH radicals was 3/4-methylphenol, with an L_OH_ range of 16.16 ± 8.85 × 10^−12^ s^−1^, accounting for 44.15% of the total ·OH radical depletion rate. Subsequently, phenol exhibited an L_OH_ value of 8.38 ± 2.84 × 10^−12^ s^−1^, representing a contribution of 22.89%. The phenolic compound with the lowest L_OH_ value was 2,4-dichlorophenol, with an L_OH_ value of 0.09 ± 0.06 × 10^−12^ s^−1^, contributing 0.25% to the total ·OH radical depletion rate. The results indicated that 3/4-methylphenol, phenol, 2,6-dimethylphenol, and 2-methylphenol were significant contributors to ·OH radical depletion in Beijing. It is noteworthy that for phenol, which exhibited the highest concentration level, the L_OH_ value and L_OH_ contribution were smaller than those of 3/4-methylphenol. This indicates that phenol contributed less to ·OH radical loss than 3/4-methylphenol, likely due to its smaller K_OH_ value (28.3 × 10^−12^).

At the Heze site, the phenolic compound with the greatest impact on OH radical depletion was phenol, with an L_OH_ value of 6.72 ± 1.81 × 10^−12^ s^−1^, accounting for 30.5%. This was followed by 3/4-methylphenol, with an L_OH_ value of 6.21 ± 2.36 × 10^−12^ s^−1^, accounting for 28.2% of the total ·OH radical depletion rate. The phenolic compound with the lowest L_OH_ value was 2,4-dichlorophenol, with a value of 0.08 s^−1^, contributing 0.2% to the total ·OH radical depletion rate.

The higher L_OH_ values of volatile phenols in Beijing relative to Heze may be attributed to elevated industrial and transportation activities in Beijing, leading to increased atmospheric concentrations of volatile phenols and accelerated reaction rates with ·OH radicals. Furthermore, it is evident that the primary species contributing to ·OH radical loss at the Heze site differs from those observed in Beijing. Given the significantly higher K_OH_ value of 3/4-methylphenol (64 × 10^−12^) compared to phenol, the latter remains the dominant volatile phenol species contributing to hydroxyl depletion at the Heze site. Prior research has indicated that the primary source of atmospheric phenol is natural, such as through the decomposition of leaves or wood [59]. This finding highlights the differences in urban structure between the Heze region, dominated by agricultural and forestry land, and the Beijing region, characterized by developed urban areas, as well as the strong influence of industrial emissions on volatile phenol emissions and photochemical activity. These factors should be emphasized in future studies.

#### 3.4.3. OFP

The quantification of hydroxyl radical depletion rates provides crucial insights into the atmospheric removal efficiency of volatile organic compounds (VOCs), while the assessment of OFP evaluates their role in ozone production. The OFP calculation results are shown in Figure 6b.

The sensitivity results of the baseline MIR’s OFP and ±20% MIR are presented in Supplementary Materials Table S3. The comparison shows that the conclusions of this study (including species relative ranking and major pollution characteristics) are robust to ±20% interference of the MIR [39,40]. In this study, the OFP values for volatile phenols during winter in Beijing were calculated as 172.74 ± 73.80 ppbv O_3_. The primary contributor to O_3_ production was phenol (59.97 ± 27.79 ppbv O_3_), accounting for 34.72%, followed by 3/4-methylphenol (55.91 ± 35.72 ppbv O_3_), contributing 32.37%. In Heze, the total OFP value was 121.04 ± 22.21 ppbv O_3_, with phenol (59.84 ± 14.21 ppbv O_3_) representing 59.84% and 3/4-methylphenol (22.94 ± 8.10 ppbv O_3_) contributing 18.95%. These findings underscore the significant contributions of phenol and 3/4-methylphenol to ozone generation at both locations, consistent with ·OH radical depletion rate observations. The higher OFP value observed in Beijing suggests a more substantial potential for volatile phenols to contribute to ozone formation under comparable VOC emission conditions.

Comparison of Beijing’s OFP values across different years reveals notable fluctuations; for instance, the winter OFP was 345.6 ppbv O_3_ in 2017, contrasting sharply with 101.9 ppbv O_3_ in 2014, indicating a distinct downward trend. Long-term observations in Hong Kong, China, also reflect declining OFP values: 474.1 μg/m^3^ O_3_ in winter 2003: http://www.td.gov.hk/, accessed on 27, April, 2025), 278.8 μg/m^3^ O_3_ in 2013 [60], and 190.8 μg/m^3^ O_3_ in 2015 [61]. Similar trends are observed in other Chinese cities such as Weinan [62] and Xi’an [54]. underscoring the evolving impact of environmental policies, notably, the 14th Five-Year Plan, on atmospheric quality across China.

Spatial variations in OFP values between urban centers highlight distinct urban structures and emissions profiles. Beijing, characterized by developed urban areas, high vehicle ownership, and intensive industrial activities, exhibits elevated VOC emissions. In contrast, Heze’s urban landscape, dominated by agriculture and light industry with lower traffic density, shows comparatively lower VOC emissions. Disparities in OFP values among cities like Xi’an (168 ppbv O_3_) [54], Wuhan (82.49 ppbvO_3_) [62], and Baoji (126 ppbv O_3_) [63] during winter 2017 reflect variations in industrial structure, transportation dynamics, and population densities.

The elevated OFP and L_OH_ values of volatile phenols underscore their substantial atmospheric reactivity and pivotal role in ozone and secondary organic aerosol (SOA) formation. This emphasizes the need for targeted control strategies addressing volatile phenol emissions in air pollution management. Future research should further investigate industrial structure disparities between Beijing and Heze, as well as the influence of winter weather conditions on volatile phenol reactivity. Such studies will enhance our understanding of volatile phenol chemistry in diverse environmental contexts, supporting the development of more effective pollution control measures.

### 3.5. Analysis of Different Sources

Positive Matrix Factorization (PMF) is a receptor model extensively employed for source apportionment, aimed at identifying pollutant source profiles and quantifying their contributions. Previous studies have demonstrated the broad applicability of PMF across various domains, including atmospheric fine particulate matter [64,65,66], hazardous heavy metals [67,68], and VOCs [69,70]. Refer to Supplementary Materials Text S1 for more details of the PMF model.

**Figure 7:**
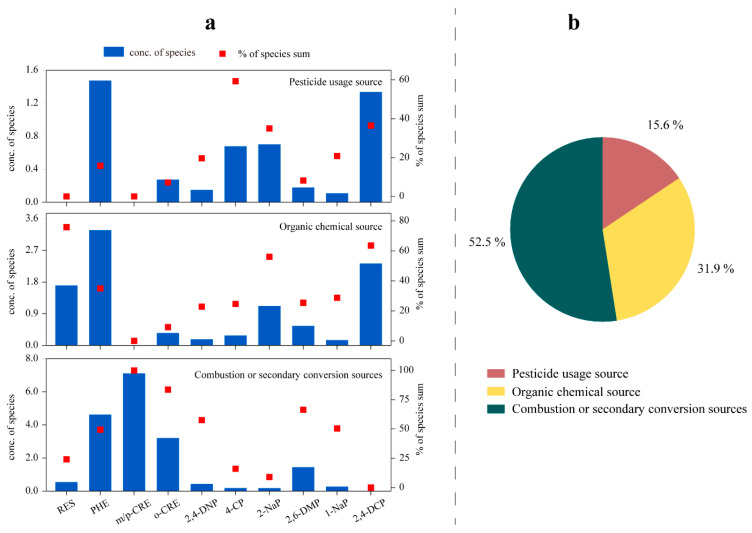
Results of fitting based on the PMF model. (**a**) Atmospheric species concentrations (blue bars) and their percentages in total species (red dots) corresponding to three pollution sources (pesticide use sources, organic chemical sources, and combustion or secondary transformation sources); (**b**) contribution percentages of various sources.

In this study, a PMF model was employed to discern the primary source factors influencing winter phenol concentrations in Beijing. These factors were identified as pesticide usage, organic chemical sources, and combustion or secondary conversion sources. The concentrations and respective contributions of the eleven phenols, as delineated by the PMF model, are visually presented in Figure 7a. Furthermore, Figure 7b illustrates the proportional distribution of the various phenol sources. It is important to note that due to the comparatively lower atmospheric phenol concentrations observed at the rural Heze site, the PMF model did not achieve precise discrimination between specific sources in that location.

The first factor prominently featured 4-chlorophenol (4-CP), constituting 59.3% of its overall contribution. 4-CP is widely utilized as a synthetic insecticide, herbicide, disinfectant, and wood preservative [16]. The degradation of pesticides yields significant quantities of chlorophenol compounds. Consequently, Factor 1 was identified as originating from pesticide usage, contributing the least at 15.6%.

The second factor was characterized by RES, 2-naphthol (2-NaP), and 2,4-dichlorophenol (2,4-DCP), contributing 75.8%, 56.1%, and 63.5%, respectively. These compounds are extensively used in various organic chemical processes, such as dye production and synthetic rubber manufacturing [31,71,72,73]. They are commonly found in phenol-containing wastewater and waste gases from diverse organic chemical industries. Hence, Factor 2 was associated with an organic chemical source, contributing 31.9%.

The third factor exhibited significant proportions of *m/p*-cresol (*m/p*-CRE), o-cresol (o-CRE), 2,6-dimethylphenol (2,6-DMP), and 2,4-dinitrophenol (2,4-DNP), contributing 100.0%, 83.6%, 66.38%, and 57.5%, respectively. These compounds are typically linked to phenolic emissions from biomass combustion [7,67] and can also result from secondary photochemical reactions involving atmospheric OH [74]. These reactions often stem from benzene and toluene, and 2,4-DNP can either be directly emitted from vehicle combustion processes or formed indirectly in the atmosphere through photochemical reactions involving precursors such as benzene, toluene, phenol, and cresol, in conjunction with ·OH and NO_x_ [75,76]. Consequently, Factor 3 was identified as a source associated with combustion or secondary conversion, accounting for the largest contribution at 52.5%. Notably, combustion or secondary conversion sources exerted a significant influence on phenol pollution in the environment.

## 4. Conclusions

In the present research, we conducted an investigation into the concentration levels, activity characteristics, and sources in the atmosphere of 11 phenolic compounds, aiming to address the significant gap in quantitative observational data on atmospheric volatile phenols in urban of Beijing and a rural locale in Heze, Shandong province. The findings revealed the following:

(1) The concentrations of volatile phenols observed in this study are notably higher compared to results obtained using PUF membranes in other studies. Given the relatively limited attention paid to volatile phenols in conventional volatile organic compounds (VOCs) research, continued emphasis on volatile phenols in atmospheric studies is warranted.

(2) Among the phenolic compounds, phenol (PHE) (6.30 ppbv), *m/p*-cresol (*m/p*-CRE) (5.98 ppbv), and o-cresol (o-CRE) (3.39 ppbv) were most abundant in Beijing, whereas higher concentrations of phenol (PHE) (5.32 ppbv) and 2-naphthol (2-NaP) (3.07 ppbv) were observed in Heze. Moreover, daily concentration trends exhibited more pronounced variations in Beijing under varying pollution conditions, characterized by an initial decline followed by subsequent increases.

(3) Compared with other non-methane hydrocarbon VOCs and aldehyde ketone OVOCs, higher level of L_OH_ for volatile phenols in Beijing was found, indicating that the activity of volatile phenols in the atmosphere and their impact on atmospheric oxidation capacity cannot be ignored.

(4) Source apportionment using the PMF model identified three primary sources of phenolic compounds: pesticide usage (15.6%), organic chemical sources (31.9%), and combustion or secondary conversion sources (52.5%).

We anticipate that the insightful findings derived from this research will significantly contribute to advancing atmospheric observations concerning volatile phenols in China. Furthermore, these findings offer crucial theoretical guidance for local efforts aimed at mitigating air pollution challenges posed by these compounds.

## Data Availability

The original contributions presented in this study are included in the article/Supplementary Material. Further inquiries can be directed to the corresponding author.

## Supplementary Materials

The following supporting information can be downloaded at: https://www.mdpi.com/article/10.3390/toxics13090744/s1, Figure S1: Sample enrichment of volatile phenols at different flow rates. RES: resorcinol; PHE: phenol; o-CRE: o-cresol; m/p-CRE: m-, p-cresol; 2,4-DNP: 2,4-dinitrophenol; 4-CP: 4-chlorophenol; 1-NaP: 1-naphthol; 2-NaP: 2-naphthol; 2,6-DMP: 2,6-dimethylphenol; 2,4-DCP: 2,4-dichlorophenol; Figure S2: Daily changes of 11 phenols in Beijing (a) and Heze (b) in winter under different pollution conditions. SADs: superior air quality days, SMP: slight—moderate pollution, HP: heavy pollution, SP: severe pollution; Figure S3: Heat map of the correlation between phenolic compounds and meteorological factors of Beijing (a) and Heze (b); Table S1: Detailed information for the 11 phenols, mainly including abbreviation, molecular formula, CAS NO., structure, co-relationship (R), method detection limit (MDL), precision (RSD), and Recovery rate; Table S2: Gas-phase OH reaction rate constants, MIR values, and ranges with ±20% uncertainty for eight phenolic compounds at 298 K; Table S3: Robustness assessment of L_OH_ and OFP (±20% K_OH_ and ±20%MIR); Table S4: Summary of LOH, OFP, and SOAFP values by location; Text S1: PMF model; Text S2: Explanation of K_OH_ value values; Text S3: Explanation of MIR value values. Refs. [62,77,78,79,80,81,82,83,84,85,86,87,88,89,90] are cited in the Supplementary Materials.

## References

[1] Guo S.J., Chen M., Tan J.H. (2016). Seasonal and diurnal characteristics of atmospheric carbonyls in Nanning, China. Atmos. Res..

[2] Mellouki A., Wallington T.J., Chen J. (2015). Atmospheric chemistry of oxygenated volatile organic compounds: Impacts on air quality and climate. Chem. Rev..

[3] Liu G., Ma X., Li W., Chen J., Ji Y., An T. (2024). Pollution characteristics, source appointment and environmental effect of oxygenated volatile organic compounds in Guangdong-Hong Kong-Macao Greater Bay Area: Implication for air quality management. Sci. Total Environ..

[4] Huang Y., Li X.R., Chen X., Wang W.J., Wang Y.H., Liu Z.R., Tang G.Q. (2022). Low-molecular-weight carbonyl volatile organic compounds on the North China Plain. Atmos. Environ..

[5] Nakao S., Clark C., Tang P., Sato K., Cocker Iii D. (2011). Secondary organic aerosol formation from phenolic compounds in the absence of NOx. Atmos. Chem. Phys..

[6] Rogge W.F., Hildemann L.M., Mazurek M.A., Cass G.R., Simoneit B.R.T. (1998). Sources of fine organic aerosol. 9. Pine, oak and synthetic log combustion in residential fireplaces. Environ. Sci. Technol..

[7] Schauer J.J., Kleeman M.J., Cass G.R., Simoneit B.R.T. (2001). Measurement of emissions from air pollution sources. 3. C_1_-C_29_ organic compounds from fireplace combustion of wood. Environ. Sci. Technol..

[8] Smith J.D., Sio V., Yu L., Zhang Q., Anastasio C. (2014). Secondary organic aerosol production from aqueous reactions of atmospheric phenols with an organic triplet excited state. Environ. Sci. Technol..

[9] Li M., Wang X., Lu C., Li R., Zhang J., Dong S., Yang L., Xue L., Chen J., Wang W. (2020). Nitrated phenols and the phenolic precursors in the atmosphere in urban Jinan, China. Sci. Total Environ..

[10] Nojima K., Isogami C., Itoh H., Kawaguchi A. (1994). Studies on Photochemical Reactions of Air Pollutants. XII. Photochemical Epoxidation of Aldrin with Suspended Particulates in Air. Biol. Pharm. Bull..

[11] Bolzacchini E., Bruschi M., Hjorth J., Meinardi S., Orlandi M., Rindone B., Rosenbohm E. (2001). Gas-phase reaction of phenol with NO_3_. Environ. Sci. Technol..

[12] Olariu R.I., Klotz K., Barnes I., Becker K.H., Mocanu R. (2002). FT-IR study of the ring-retaining products from the reaction of OH radicals with phenol, o-, m-, and p-cresol. Atmos. Environ..

[13] Wang Y., Hu M., Wang Y., Zheng J., Shang D., Yang Y., Liu Y., Li X., Tang R., Zhu W. (2019). The formation of nitro-aromatic compounds under high NO_x_ and anthropogenic VOC conditions in urban Beijing, China. Atmos. Chem. Phys..

[14] Ji Y.M., Zhao J., Terazono H., Misawa K., Levitt N.P., Li Y.X., Lin Y., Peng J.F., Wang Y., Duan L. (2017). Reassessing the atmospheric oxidation mechanism of toluene. Proc. Natl. Acad. Sci. USA.

[15] Liang Y., Wang X., Dong S., Liu Z., Mu J., Lu C., Zhang J., Li M., Xue L., Wang W. (2020). Size distributions of nitrated phenols in winter at a coastal site in north China and the impacts from primary sources and secondary formation. Chemosphere.

[16] Shen W., Mu Y., Wang B., Ai Z., Zhang L. (2017). Enhanced aerobic degradation of 4-chlorophenol with iron-nickel nanoparticles. Appl. Surf. Sci..

[17] Wright J.S., Shadnia H. (2008). Computational Modeling of Substituent Effects on Phenol Toxicity. Chem. Res. Toxicol..

[18] Atkinson R., Aschmann S.M., Arey J. (2002). Reactions of hydroxyl and nitrogen trioxide radicals with phenol, cresols, and 2-nitrophenol at 296 ± 2 K. Environ. Sci. Technol..

[19] Fang Z., Lai A., Dongmei C., Chunlin L., Carmieli R., Chen J., Wang X., Rudich Y. (2024). Secondary Organic Aerosol Generated from Biomass Burning Emitted Phenolic Compounds: Oxidative Potential, Reactive Oxygen Species, and Cytotoxicity. Environ. Sci. Technol..

[20] Li C., He Q., Hettiyadura A.P.S., Käfer U., Shmul G., Meidan D., Zimmermann R., Brown S.S., George C., Laskin A. (2020). Formation of Secondary Brown Carbon in Biomass Burning Aerosol Proxies through NO_3_ Radical Reactions. Environ. Sci. Technol..

[21] Ogrizek M., Kroflic A., Sala M. (2022). Determination of trace concentrations of simple phenols in ambient PM samples. Chemosphere.

[22] Yu L., Smith J., Laskin A., George K.M., Anastasio C., Laskin J., Dillner A.M., Zhang Q. (2016). Molecular transformations of phenolic SOA during photochemical aging in the aqueous phase: Competition among oligomerization, functionalization, and fragmentation. Atmos. Chem. Phys..

[23] Li X., Zhao Q., Yang Y., Zhao Z., Liu Z., Wen T., Hu B., Wang Y., Wang L., Wang G. (2021). Composition and sources of brown carbon aerosols in megacity Beijing during the winter of 2016. Atmos. Res..

[24] Yang Y., Li X., Shen R., Liu Z., Ji D., Wang Y. (2020). Seasonal variation and sources of derivatized phenols in atmospheric fine particulate matter in North China Plain. J. Environ. Sci..

[25] Li X., Yang Y., Liu S., Zhao Q., Wang G., Wang Y. (2020). Light absorption properties of brown carbon (BrC) in autumn and winter in Beijing: Composition, formation and contribution of nitrated aromatic compounds. Atmos. Environ..

[26] Delhomme O., Morville S., Millet M. (2010). Seasonal and diurnal variations of atmospheric concentrations of phenols and nitrophenols measured in the Strasbourg area, France. Atmos. Pollut. Res..

[27] Mayorga R.J., Zhao Z., Zhang H. (2021). Formation of secondary organic aerosol from nitrate radical oxidation of phenolic VOCs: Implications for nitration mechanisms and brown carbon formation. Atmos. Environ..

[28] Liu C., Chen D., Chen X.E. (2022). Atmospheric Reactivity of Methoxyphenols: A Review. Environ. Sci. Technol..

[29] Li F., Zhou S., Du L., Zhao J., Hang J., Wang X. (2023). Aqueous-phase chemistry of atmospheric phenolic compounds: A critical review of laboratory studies. Sci. Total Environ..

[30] Mao L., Chen H., Liu G., Peng Z., Chen W., Kang L., Liao S. (2011). Flow injection analysis method for hygienic examination of volatile phenol compounds in the air of residential area. J. Hyg. Res..

[31] Huang X., Zhao G., Liu M., Li F., Qiao J., Zhao S. (2012). Highly sensitive electrochemical determination of 1-naphthol based on high-index facet SnO_2_ modified electrode. Electrochim. Acta.

[32] Belloli R., Barletta B., Bolzacchini E., Meinardi S., Orlandi M., Rindone B. (1999). Determination of toxic nitrophenols in the atmosphere by high-performance liquid chromatography. J. Chromatogr. A.

[33] Cecinato A., Palo V.D., Pomata D., Sciano M.C.T., Possanzini M. (2005). Measurement of phase-distributed nitrophenols in Rome ambient air. Chemosphere.

[34] Chen Y., Li W., Jia R. (2017). Separation and determination of phenolic compounds in ambient air by ultra-high performance liquid chromatography. Appl. Chem. Ind..

[35] Liu Y., Yin S., Zhang S., Ma W., Zhang X., Qiu P., Li C., Wang G., Hou D., Zhang X. (2024). Drivers and impacts of decreasing concentrations of atmospheric volatile organic compounds (VOCs) in Beijing during 2016–2020. Sci. Total Environ..

[36] Zhang Y., Li R., Fu H., Zhou D., Chen J. (2018). Observation and analysis of atmospheric volatile organic compounds in a typical petrochemical area in Yangtze River Delta, China. J. Environ. Sci..

[37] Atkinson R., Arey J. (2003). Atmospheric Degradation of Volatile Organic Compounds. Chem. Rev..

[38] Atkinson R., Baulch D.L., Cox R.A., Crowley J.N., Hampson R.F., Hynes R.G., Jenkin M.E., Rossi M.J., Troe J., Subcommittee I. (2006). Evaluated kinetic and photochemical data for atmospheric chemistry: Volume II &ndash; gas phase reactions of organic species. Atmos. Chem. Phys..

[39] Zhang Y., Xue L., Mu J., Chen T., Li H., Gao J., Wang W. (2022). Developing the Maximum Incremental Reactivity for Volatile Organic Compounds in Major Cities of Central-Eastern China. J. Geophys. Res. Atmos..

[40] Venecek M.A., Carter W.P.L., Kleeman M.J. (2018). Updating the SAPRC Maximum Incremental Reactivity (MIR) scale for the United States from 1988 to 2010. J. Air Waste Manag. Assoc..

[41] Carter W. (2010). Updated Maximum Incremental Reactivity Scale and Hydrocarbon Bin Reactivities for Regulatory Applications.

[42] Rubio M.A., Lissi E., Herrera N., Pérez V., Fuentes N. (2012). Phenol and nitrophenols in the air and dew waters of Santiago de Chile. Chemosphere.

[43] Sreekanth R., Prasanthkumar K.P., Sunil Paul M.M., Aravind U.K., Aravindakumar C.T. (2013). Oxidation Reactions of 1- and 2-Naphthols: An Experimental and Theoretical Study. J. Phys. Chem. A.

[44] Sun J.F., Mu Q., Kimura H., Murugadoss V., He M.X., Du W., Hou C.X. (2022). Oxidative degradation of phenols and substituted phenols in the water and atmosphere: A review. Adv. Compos. Hybrid Mater..

[45] Song M., Tan Q., Feng M., Qu Y., Liu X., An J., Zhang Y. (2018). Source Apportionment and Secondary Transformation of Atmospheric Nonmethane Hydrocarbons in Chengdu, Southwest China. J. Geophys. Res. Atmos..

[46] Zou Y., Deng X.J., Zhu D., Gong D.C., Wang H., Li F., Tan H.B., Deng T., Mai B.R., Liu X.T. (2015). Characteristics of 1 year of observational data of VOCs, NOx and O_3_ at a suburban site in Guangzhou, China. Atmos. Chem. Phys..

[47] Deng Y., Li J., Li Y., Wu R., Xie S. (2019). Characteristics of volatile organic compounds, NO_2_, and effects on ozone formation at a site with high ozone level in Chengdu. J. Environ. Sci..

[48] Kutuzov S., Legrand M., Preunkert S., Ginot P., Mikhalenko V., Shukurov K., Poliukhov A., Toropov P. (2019). The Elbrus (Caucasus, Russia) ice core record—Part 2: History of desert dust deposition. Atmos. Chem. Phys..

[49] Huang W., Kuzyakov Y., Niu S., Luo Y., Sun B., Zhang J., Liang Y. (2023). Drivers of microbially and plant-derived carbon in topsoil and subsoil. Glob. Chang. Biol..

[50] Valanciene E., Jonuskiene I., Syrpas M., Augustiniene E., Matulis P., Simonavicius A., Malys N. (2020). Advances and Prospects of Phenolic Acids Production, Biorefinery and Analysis. Biomolecules.

[51] Song K., Guo S., Wang H., Yu Y., Wang H., Tang R., Xia S., Gong Y., Wan Z., Lv D. (2021). Measurement report: Online measurement of gas-phase nitrated phenols utilizing a CI-LToF-MS: Primary sources and secondary formation. Atmos. Chem. Phys..

[52] Yuan B., Liggio J., Wentzell J., Li S.M., Stark H., Roberts J.M., Gilman J., Lerner B., Warneke C., Li R. (2016). Secondary formation of nitrated phenols: Insights from observations during the Uintah Basin Winter Ozone Study (UBWOS) 2014. Atmos. Chem. Phys..

[53] Zhang X., Kong Y., Cao J., Li H., Gao R., Zhang Y., Wang K., Li Y., Ren Y., Wang W. (2022). A sensitive simultaneous detection approach for the determination of 30 atmospheric carbonyls by 2,4-dinitrophenylhydrazine derivatization with HPLC-MS technique and its preliminary application. Chemosphere.

[54] Li J., Deng S., Tohti A., Li G., Yi X., Lu Z., Liu J., Zhang S. (2022). Spatial characteristics of VOCs and their ozone and secondary organic aerosol formation potentials in autumn and winter in the Guanzhong Plain, China. Environ. Res..

[55] Liu T., Hong Y., Li M., Xu L., Chen J., Bian Y., Yang C., Dan Y., Zhang Y., Xue L. (2022). Atmospheric oxidation capacity and ozone pollution mechanism in a coastal city of southeastern China: Analysis of a typical photochemical episode by an observation-based model. Atmos. Chem. Phys..

[56] Guan Y., Zhang Y., Zhang Y., Wang X., Han J., Song W., Hou L.A., Duan E. (2020). Pollution Characteristics and Key Reactive Species of Volatile Organic Compounds in Beijing-Tianjin-Hebei Area, China. Aerosol Air Qual. Res..

[57] Zhang R., Lu H., Deng S., Rui S., Wang W. (2019). Characteristics of VOCs and formation potential of O, and SOA in autumn and winter in Baoii, China. China Environ. Sci..

[58] Luo H., Chen J., Li G., An T. (2021). Formation kinetics and mechanisms of ozone and secondary organic aerosols from photochemical oxidation of different aromatic hydrocarbons: Dependence on NOx and organic substituents. Atmos. Chem. Phys..

[59] Mahugo S.C., Sosa F.Z., Esther T.P.M., Juan S., Rodriguez J. (2009). Methodologies for the extraction of phenolic compounds from environmental samples: New approaches. Molecules.

[60] Han S., Tan Y., Gao Y., Li X., Ho S.S.H., Wang M., Lee S.C. (2023). Volatile organic compounds at a roadside site in Hong Kong: Characteristics, chemical reactivity, and health risk assessment. Sci. Total Environ..

[61] Cui L., Wang X.L., Ho K.F., Gao Y., Liu C., Hang Ho S.S., Li H.W., Lee S.C., Wang X.M., Jiang B.Q. (2018). Decrease of VOC emissions from vehicular emissions in Hong Kong from 2003 to 2015: Results from a tunnel study. Atmos. Environ..

[62] Hui L., Liu X., Tan Q., Feng M., An J., Qu Y., Zhang Y., Jiang M. (2018). Characteristics, source apportionment and contribution of VOCs to ozone formation in Wuhan, Central China. Atmos. Environ..

[63] Xue Y., Ho S.S.H., Huang Y., Li B., Wang L., Dai W., Cao J., Lee S. (2017). Source apportionment of VOCs and their impacts on surface ozone in an industry city of Baoji, Northwestern China. Sci. Rep..

[64] Contini D., Cesari D., Conte M., Donateo A. (2016). Application of PMF and CMB receptor models for the evaluation of the contribution of a large coal-fired power plant to PM10 concentrations. Sci. Total Environ..

[65] Feng X., Feng Y., Chen Y., Cai J., Li Q., Chen J. (2022). Source apportionment of PM_2.5_ during haze episodes in Shanghai by the PMF model with PAHs. J. Clean. Prod..

[66] Zhang Z., Xu B., Xu W., Wang F., Gao J., Li Y., Li M., Feng Y., Shi G. (2022). Machine learning combined with the PMF model reveal the synergistic effects of sources and meteorological factors on PM_2.5_ pollution. Environ. Res..

[67] Liu L., Xu X., Han J., Zhu J., Li S., Liang L., Wu P., Wu Q., Qiu G. (2022). Heavy metal(loid)s in agricultural soils in the world’s largest barium-mining area: Pollution characteristics, source apportionment, and health risks using PMF model and Cd isotopes. Process Saf. Environ. Prot..

[68] Magesh N.S., Tiwari A., Botsa S.M., da Lima Leitao T. (2021). Hazardous heavy metals in the pristine lacustrine systems of Antarctica: Insights from PMF model and ERA techniques. J. Hazard. Mater..

[69] Huang A., Yin S., Yuan M., Xu Y., Yu S., Zhang D., Lu X., Zhang R. (2022). Characteristics, source analysis and chemical reactivity of ambient VOCs in a heavily polluted city of central China. Atmos. Pollut. Res..

[70] Li Y., Gao R., Xue L., Wu Z., Yang X., Gao J., Ren L., Li H., Ren Y., Li G. (2021). Ambient volatile organic compounds at Wudang Mountain in Central China: Characteristics, sources and implications to ozone formation. Atmos. Res..

[71] Krugly E., Martuzevicius D., Tichonovas M., Jankunaite D., Rumskaite I., Sedlina J., Racys V., Baltrusaitis J. (2015). Decomposition of 2-naphthol in water using a non-thermal plasma reactor. Chem. Eng. J..

[72] Porras S.P., Hartonen M., Ylinen K., Tornaeus J., Tuomi T., Santonen T. (2018). Environmental and occupational exposure to resorcinol in Finland. Toxicol. Lett..

[73] Rao Q., Hu G., Zhang C., Yang H., Hu F., Guo C. (2023). Electrochemical Sensor Construction of Carbon-based Materials for Ultrasensitive and Precise Determination of Dihydroxybenzene Isomers:a Review. Mater. Rev..

[74] Pillar E.A., Guzman M.I. (2017). Oxidation of Substituted Catechols at the Air-Water Interface: Production of Carboxylic Acids, Quinones, and Polyphenols. Environ. Sci. Technol..

[75] Morville S., Scheyer A., Mirabel P., Millet M. (2004). A multiresidue method for the analysis of phenols and nitrophenols in the atmosphere. J. Environ. Monit..

[76] Tremp J., Mattrel P., Fingler S., Giger W. (1993). Phenols and nitrophenols as tropospheric pollutants: Emissions from automobile exhausts and phase transfer in the atmosphere. Water Air Soil Pollut..

[77] Zhang C., Liu X., Zhang Y., Tan Q., Feng M., Qu Y., An J., Deng Y., Zhai R., Wang Z. (2021). Characteristics, source apportionment and chemical conversions of VOCs based on a comprehensive summer observation experiment in Beijing. Atmos. Pollut. Res..

[78] Berndt T., Böge O. (2003). Gas-phase reaction of OH radicals with phenol. Phys. Chem. Chem. Phys..

[79] Berg F., Novelli A., Dubus R., Hofzumahaus A., Holland F., Wahner A., Fuchs H. (2024). Temperature-dependent rate coefficients for the reactions of OH radicals with selected alkanes, aromatic compounds, and monoterpenes. Atmos. Chem. Phys..

[80] Semadeni M., Stocker D.W., Kerr J.A. (2004). The temperature dependence of the OH radical reactions with some aromatic compounds under simulated tropospheric conditions. Int. J. Chem. Kinet..

[81] Han L., Siekmann F., Zetzsch C. (2018). Rate Constants for the Reaction of OH Radicals with Hydrocarbons in a Smog Chamber at Low Atmospheric Temperatures. Atmosphere.

[82] Li J., Deng S., Li G., Lu Z., Song H., Gao J., Sun Z., Xu K. (2022). VOCs characteristics and their ozone and SOA formation potentials in autumn and winter at Weinan, China. Environ. Res..

[83] Wang S., Zhao Y., Han Y., Li R., Fu H., Gao S., Duan Y., Zhang L., Chen J. (2022). Spatiotemporal variation, source and secondary transformation potential of volatile organic compounds (VOCs) during the winter days in Shanghai, China. Atmos. Environ..

[84] Wang G., Cheng S., Wei W., Zhou Y., Yao S., Zhang H. (2016). Characteristics and source apportionment of VOCs in the suburban area of Beijing, China. Atmos. Pollut. Res..

[85] Kalbande R., Yadav R., Maji S., Rathore D.S., Beig G. (2022). Characteristics of VOCs and their contribution to O_3_ and SOA formation across seasons over a metropolitan region in India. Atmos. Pollut. Res..

[86] Yi X.X., Li J.H., Li G.H., Lu Z.Z., Sun Z.G., Gao J., Deng S.X. (2022). Characteristics of VOCs and Formation Potentials of O_3_ and SOA in Autumn and Winter in Tongchuan, China. Huan Jing Ke Xue.

[87] Liu X., Lu J., Li W., Liu Z., Tong Y., Chen H., Yu J., Ding Y. (2022). Characterization, source apportionment, and assessment of volatile organic compounds in a typical urban area of southern Xinjiang, China. Air Qual. Atmos. Health.

[88] Jia C., Mao X., Huang T., Liang X., Wang Y., Shen Y., Jiang W., Wang H., Bai Z., Ma M. (2016). Non-methane hydrocarbons (NMHCs) and their contribution to ozone formation potential in a petrochemical industrialized city, Northwest China. Atmos. Res..

[89] Wang C., Huang X.F., Han Y., Zhu B., He L.Y. (2017). Sources and Potential Photochemical Roles of Formaldehyde in an Urban Atmosphere in South China. J. Geophys. Res. Atmos..

[90] Shirley T.R., Brune W.H., Ren X., Mao J., Lesher R., Cardenas B., Volkamer R., Molina L.T., Molina M.J., Lamb B. (2006). Atmospheric oxidation in the Mexico City Metropolitan Area (MCMA) during April 2003. Atmos. Chem. Phys..

