# Supplementary material for: Investigation on the Concentration, Sources, and Photochemical Roles of Volatile Phenols in the Atmosphere in the North China Plain
Source: Toxics. 2025 Aug 31;13(9):744. doi: 10.3390/toxics13090744 (PMC12473939; doi:10.3390/toxics13090744)
Supplement: Supplementary file 1 [file toxics-13-00744-s001.zip › toxics-3791667-supplementary.pdf]

Supplementary materials

Text A1: PMF model.

Text A2: Explanation of K_{OH} value values.
Text A3: Explanation of MIR value values.
Figure S1: Sample enrichment of volatile phenols at different flow rates. RES: resorcinol; PHE: phenol; o-CRE: o-cresol; m/p-CRE: m-, p-cresol; 2,4-DNP: 2,4-dinitrophenol; 4-CP: 4-chlorophenol; 1-NaP: 1-naphthol; 2-NaP: 2-naphthol; 2,6-DMP: 2,6-dimethylphenol; 2,4-DCP: 2,4-dichlorophenol.

Figure S2: Daily changes of 11 phenols in Beijing (a) and Heze (b) in winter under different pollution conditions. SADs: superior air quality days, SMP: slight - moderate pollution, HP: heavy pollution, SP: severe pollution.

Figure S3: Heat map of the correlation between phenolic compounds and meteorological factors of Beijing (a) and Heze (b).

Table S1: Detailed information for the 11 phenols, mainly including abbreviation, molecular formula, CAS NO., structure, co-relationship (R), method detection limit (MDL), precision (RSD), and Recovery rate.

Table S2: Gas-phase OH reaction rate constants, MIR values, and ranges with ±20% uncertainty for eight phenolic compounds at 298 K.

Table S3: Robustness assessment of L_{OH} and OFP (±20% K_{OH} and ±20%MIR).

Table S4: Summary of L_{OH}, OFP, and SOAFP values by location.

Text A1: PMF model.

For the purpose of being able to identify and evaluate each resolved factor to make its physical meaning sensible, the sample statistical matrix is first divided into two matrices of factor contributions (G) and factor profiles (F) with non-negative restrictions. The EPA PMF 5.0 model was used in this study to analyze the main sources of phenols. The basic algorithm is as follows [1-4]:

$$x_{ij} = \sum_{k=1}^{p} g_{ik} f_{kj} + e_{ij} \quad (1)$$

where x_{ij} is the concentration of substance j in sample i; p is the number of factors, i.e., the number of sources; g_{ik} is the factor spectrum, i.e., it represents the relative contribution of sample i in the kth factor (source); f_{kj} is the source contribution, i.e., the amount of substance j in the k-factor composition spectrum; and e_{ij} is the residuals of substance j of sample i in the PMF calculation process. The sample concentration and its related uncertainty are needed by the PMF so as to weight the points. The function Q is minimized by the PMF model:

$$Q = \sum_{i=1}^{n} \sum_{j=1}^{m} \left[\frac{x_{ij} - \sum_{k=1}^{p} g_{ik} f_{kj}}{u_{ij}} \right] \quad (2)$$

The EPA PMF 5.0 model's parsing process deleted missing data. Substance concentrations below the MDL are used to calculate the uncertainty using the fixed fraction of MDL (Eq. 5). Concentrations above the MDL, the uncertainty is calculated based on the concentration and MDL fraction (Eq. 6).

$$Unc = \frac{5}{6} \times MDL \quad (3)$$

$$Unc = \sqrt{(ErrorFraction \times concentration)^2 + (0.5 \times MDL)^2} \quad (4)$$

Text A2 Explanation of K_{OH} value values

Since K_{OH} changes at different temperatures, L_{OH} data will indeed produce a certain degree of deviation. According to the Arrhenius equation, the K_{OH} values at different temperatures are calculated as follows (Eq5):

$$k(T) = A\left(\frac{T}{298}\right)^{n} \exp\left(-\frac{E_a}{RT}\right) \quad (5)$$

In terms of reaction rate constants, we first collected the reaction rate constant K298 data for the target compound and hydroxyl radicals (OH), mainly from the IUPAC, NIST reaction rate databases, and related reviews. For o-cresol, we used the temperature expression Eq(6) given in the NIST (https://www.nist.gov/) entry：

$$k(T) = 2.27 \times 10^{-13}\left(\frac{T}{298}\right)^{-0.11} exp\left(\frac{1854}{RT}\right) \quad (6)$$

Unfortunately, for most phenolic and nitrophenolic compounds (especially rare compounds or those containing strong electron-withdrawing groups, such as 2,4-DNP), complete Arrhenius parameters (i.e., pre-exponential factor A, temperature exponent n, and activation energy Ea) are not available in the public literature, making it difficult to accurately calculate the temperature dependence of K_{OH} using the Arrhenius equation.

Therefore, in the absence of detailed parameters, we adopt a simplified approximation: K(T) ≈ K_{298}, i.e., assuming that the temperature dependence of the OH reaction rate constant for this class of substances is weak, with only minor adjustments made near 298 K. Previous studies have shown that even when considering typical temperature sensitivity, the change in the reaction rate constant between phenols and OH within the 280–300 K range typically does not exceed 20% [5-7]. For the main OH addition pathway of aromatic compounds, temperature dependence is generally weak; studies by Semadeni et al. and subsequent reviews support this, implying that the change in K(T) is limited within the range from 271–284 K to 298 K [8,9].

The "typical monthly average temperatures" for the observation months and locations mentioned in the article (Beijing in December/March; Heze in December/March) are approximately 271 K, 278 K, 274 K, and 284 K, respectively (see WeatherSpark and other climate annual values, https://weatherspark.com/), with deviations from 298 K being less than 27 K. Under the assumption of "weak temperature dependence," approximating k(T) as k(298) keeps the introduced systematic error within approximately 10–20%. Therefore, the approximations used in this study do not significantly affect the results of the comparative analysis, and the uncertainties are explained in the text. The above content is provided in the supplementary materials, where more detailed explanations are given.

However, based on uncertainty analysis considerations, we performed a KOH perturbation analysis with a range of ±20% on the L_{OH} and OFP data in the original manuscript. We then compared the differences between $L_{OH,298k}$ and $L_{OH,T}$. Analytically, we have:

$$\frac{\Delta L_{OH}}{L_{OH}} = \frac{\Delta K}{K}$$

Since the upper limit of temperature sensitivity is 20%, a Monte Carlo simulation (N=2×10^5) of a uniform distribution U(−20%, +20%) was performed using Python tools to quantify the statistical distribution. Under the above definition, when $\Delta\% \equiv \frac{(L_{OH,T}-L_{OH,298k})}{L_{OH,T}} \times 100\%$, mean |Δ%| ≈ 9.98%, P95 ≈ 19.00%, consistent with the analytical conclusions, this indicates that under reasonable literature uncertainty (k ±20%), temperature extrapolation typically results in L_{OH} changes of no more than 20%, with some species (such as phenol and methylphenol) showing differences as low as 10%.

If we further incorporate the uncertainty of [OH] (±20%) into the comparison of the two scenarios (representing different environmental conditions), then mean |Δ%| ≈ 13.40%, P95 ≈ 30.98%. This difference stems from environmental scenario variations rather than temperature sensitivity

itself, but the range of perturbation remains within reasonable limits. Therefore, while temperature correction is technically necessary, we believe that using the rate constant at 298K will not affect the overall scientific conclusions of this paper due to the aforementioned unavoidable factors.

Text S3 Explanation of MIR value values.

The maximum incremental reactivity (MIR) values used to calculate the OFP are derived from systematic calculations and compilations by Carter et al [10]. For representative urban scenarios under SAPRC-type photochemical mechanisms, strictly speaking, the MIR is obtained through photochemical box/ scenario simulations under defined background NOx/VOC ratios, initial concentration spectra, and meteorological conditions. To obtain "site-specific" MIR values, the same chemical mechanism (e.g., SAPRC or MCM) should be used to recalculate the MIR using the site's hourly background chemical state, emission characteristics, and meteorological drivers (i.e., run OBM/box model or regional CTM), as MIR is sensitive to model input conditions

In this paper, due to the lack of complete hourly on-site input data (including hourly NOx, VOC speciation, light intensity, and emission timing) required to accurately reconstruct the on-site box model scenario, we used the MIR values from Carter (and the CARB-published table) as baseline values for OFP estimation (the sources of MIR values for each species are listed in Supplementary Table Sx). To quantify the uncertainty associated with using the baseline MIR, we applied ±20% sensitivity perturbations to the MIR based on the literature findings regarding updates to MIR under representative conditions, and recalculated the corresponding OFP values for comparison with the baseline results (results shown in Table A3). The literature indicates that updating the representative MIR scenario from 1988 to 2010 urban conditions results in an average decrease of approximately 20% in the median MIR values across several cities [11], suggesting that using different

representative atmospheric scenarios or time periods can affect the absolute value of MIR by approximately 20%, but the relative ranking of VOCs typically remains consistent (i.e., the qualitative conclusions used for source apportionment remain robust). In previous studies, it has become standard practice to calculate winter OFP using the MIR values provided by Carter et al. under 298 K conditions [10], and the selection of these values here also facilitates parallel comparisons across different studies.

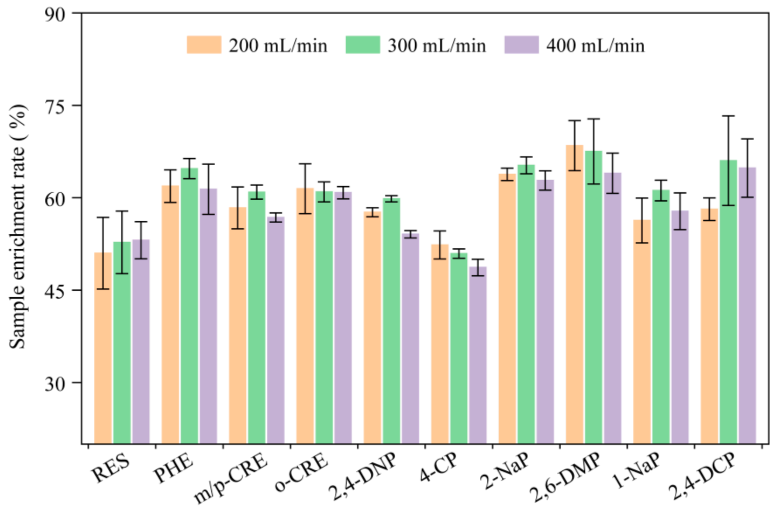

Figure S1: Sample enrichment of volatile phenols at different flow rates. RES: resorcinol; PHE: phenol; o-CRE: o-cresol; m/p-CRE: m-, p-cresol; 2,4-DNP: 2,4-dinitrophenol; 4-CP: 4-chlorophenol; 1-NaP: 1-naphthol; 2-NaP: 2-naphthol; 2,6-DMP: 2,6-dimethylphenol; 2,4-DCP: 2,4-dichlorophenol.

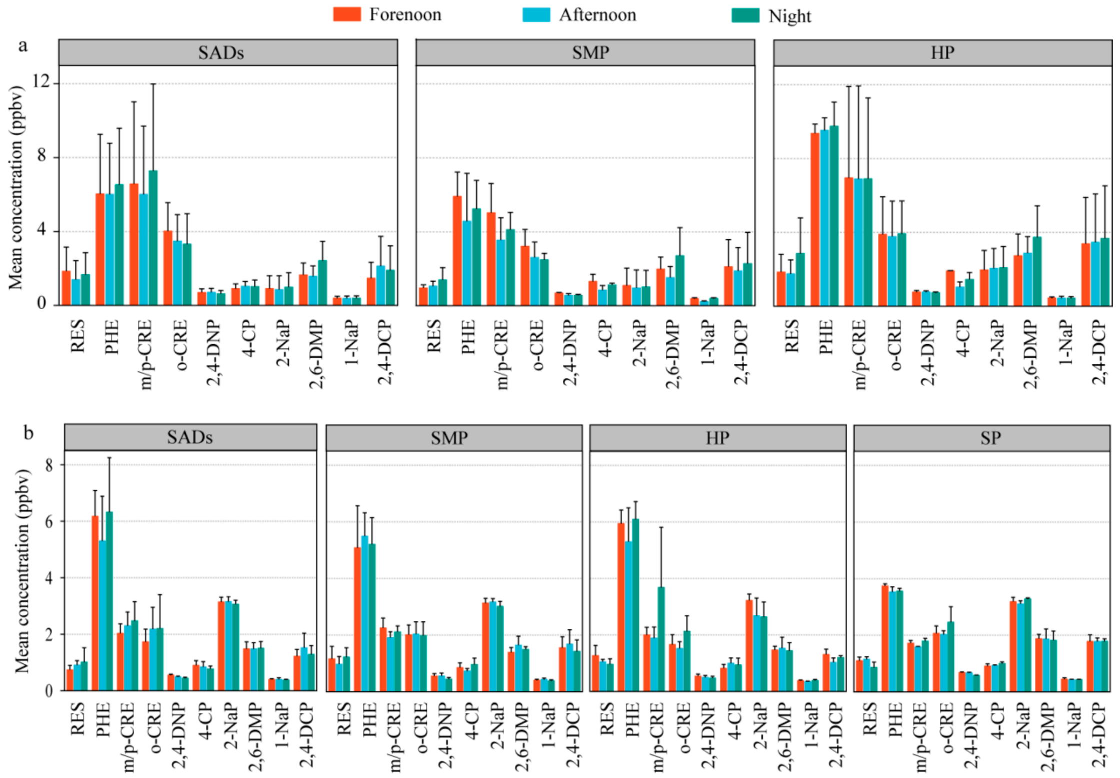

Figure S2: Daily changes of 11 phenols in Beijing (a) and Heze (b) in winter under different pollution conditions. SADs: superior air quality days, SMP: slight - moderate pollution, HP: heavy pollution, SP: severe pollution.

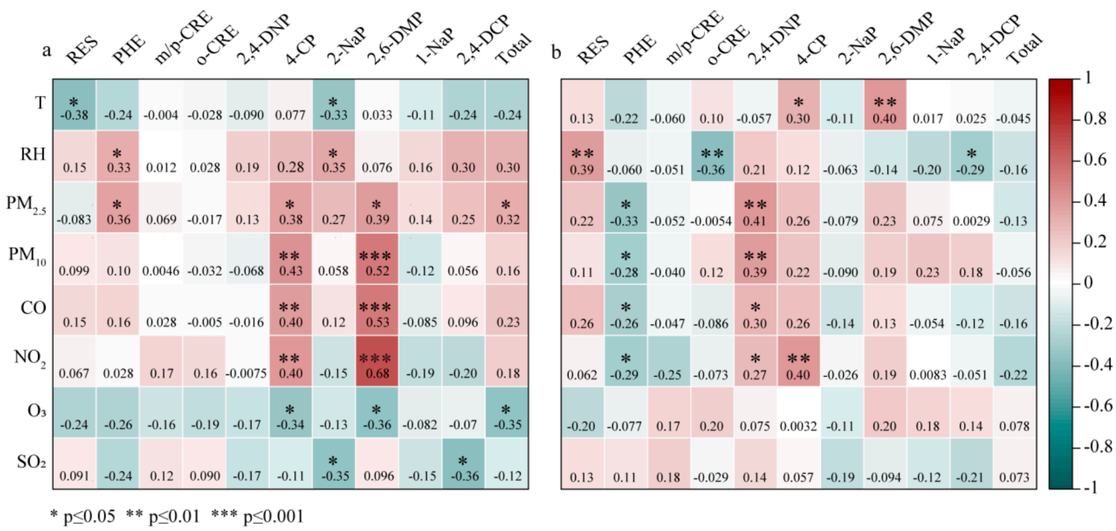

Figure S3: Heat map of the correlation between phenolic compounds and meteorological factors of Beijing (a) and Heze (b).

Table S1: Detailed information for the 11 phenols, mainly including abbreviation, molecular formula, CAS NO., structure, co-relationship (R), method detection limit (MDL), precision (RSD), and Recovery rate.

Compound	Abbreviation	Molecular formula	CAS NO.	Structure	R	MDL (ppbv)	RSD (%)	Recovery rate (%)
resorcinol	RES	$C_6H_6O_2$	108-46-3	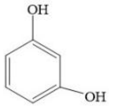	0.9993	0.49	6.46	110.4
phenol	PHE	C_6H_6O	108-95-2	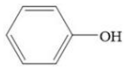	0.9991	0.69	5.65	113.1
m- and *p*-cresol	*m*/*p*-CRE	C_7H_8O	108-38-4 106-44-5	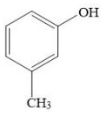 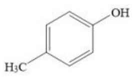	0.9994	0.67	3.84	104.4
o-cresol	o-CRE	C_7H_8O	95-48-7	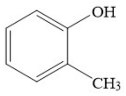	0.9995	0.62	5.96	109.9
2,4-dinitrophenol	2,4-DNP	$C_6H_4N_2O_5$	51-28-5	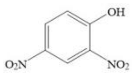	0.9995	0.15	2.99	112.4
4-chlorophenol	4-CP	C_6H_5ClO	106-48-9	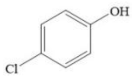	0.9994	0.40	7.31	96.6
2-naphthol	2-NaP	$C_{10}H_8O$	135-19-3	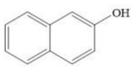	0.9994	0.25	6.90	104.5
2,6-dimethylphenol	2,6-DMP	$C_8H_{10}O$	576-26-1	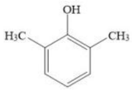	0.9996	0.50	8.01	96.9
1-naphthol	1-NaP	$C_{10}H_8O$	90-15-3	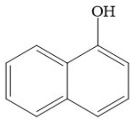	0.9994	0.22	3.80	97.7
2,4-dichlorophenol	2,4-DCP	$C_6H_4Cl_2O$	120-83-2	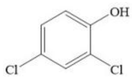	0.9993	0.29	7.51	126.2

Table S2: Gas-phase OH reaction rate constants, MIR values, and ranges with ±20% uncertainty for eight phenolic compounds at 298 K.

Compound	K_{OH} (298K) ($cm^3 \cdot molecule^{-1}\ s^{-1}$)	K_{OH} (±20%) ($cm^3 \cdot molecule^{-1}\ s^{-1}$)	MIR (298K) (g O_3/g VOC)	MIR (±20%) (g O_3/g VOC)
PHE	2.63×10^{-11}	$2.10 \sim 3.16 \times 10^{-11}$	2.69	2.15 – 3.23
m/*p*-CRE	6.4×10^{-11}	$5.12 \sim 7.68 \times 10^{-11}$	2.34	1.87 – 2.81
o-CRE	4.2×10^{-11}	$3.36 \sim 5.04 \times 10^{-11}$	2.34	1.87 – 2.81
2,4-DNP	1.7×10^{-11}	$1.36 \sim 2.04 \times 10^{-11}$	-	-
2,6-DMP	6.6×10^{-11}	$5.28 \sim 7.92 \times 10^{-11}$	2.07	1.66 – 2.48
2,4-DCP	1.06×10^{-12}	$0.85 \sim 1.27 \times 10^{-12}$	-	-
1-Nap	-	-	0.6	0.48 – 0.72

Table S3: Robustness assessment of L_{OH} and OFP (±20% K_{OH} and ±20%MIR).

Compound	L_{OH} (298K) (s^{-1})		L_{OH} (±20%) (s^{-1})		OFP (298K) (g O_3)		OFP (±20%) (g O_3)	
	BJ	HZ	BJ	HZ	BJ	HZ	BJ	HZ
PHE	7.65	7.45	6.12~9.18	5.96~8.93	59.97	59.84	47.98~71.97	47.87~71.81
m/*p*-CRE	17.09	6.72	13.67~20.51	5.38~8.06	55.91	22.94	44.73~67.09	18.35~27.53
o-CRE	6.72	4.55	5.37~8.06	3.64~5.46	38.45	22.37	30.76~46.14	17.89~26.84
2,4-DNP	1.22	0.49	0.98~1.46	0.39~0.59	10.72	9.26	10.72~10.72	7.41~11.11
2,6-DMP	6.49	5.52	5.19~7.79	4.42~6.63	-	-	-	-
2,4-DCP	-	-	-	-	7.63	6.58	6.10~9.15	5.26~7.89
1-Nap	0.43	0.42	0.34~0.51	0.33~0.50	-	-	-	-

Table S4: Summary of L_{OH}, OFP, and SOAFP values by location.

Site	Campaign time	Season	Species	L_{OH}	OFP	SOAFP	Source
Weinan(WN)	2017/11/27 - 2017/12/8	Winter	VOCs / OVOCs	20	283	149	[12]
Xi'an(XA)	2017 /11/14 -2018/1/19	Winter	VOCs / OVOCs	20.2	168	175	[13]
Shanghai(SH)	2019/12/1 - 2019/12/31	Winter	VOCs	6.98	69.72	19.32	[14]
Beijing(BJ)	2014/11/1 - 2014/11/25	Winter	VOCs		101.9		[15]
Wuhan(WH)	2016/9 - 2017/8	Annual mean	VOCs / OVOCs	5.47	82.49		[16]
Xiamen(XM)		Spring	VOCs	18.6			[17]
Hongkong(HK)	2013/11/1 -2013/11/30	Winter	VOCs / OVOCs		278.78	10.53	[18]
Pune Metropolis	2019/11 - 2019-12	Winter	VOCs		43.52	1.18	[19]
Tongchuan (TC)	2018/1/9 - 2018-1-18	Winter	VOCs / OVOCs		137.12	0.612	[20]

Baoji (BJ)	2017/12	Winter	VOCs / OVOCs	10.3	126	0.65	[21]
Xinjiang(XJ)	2019/11 - 2019/12	Winter	VOCs / OVOCs		119.84	927.61	[22]
Tangshan(TS)	2018/12/1-2018/12/31<br>2019/3/1 - 2019/3/31<br>2019/7/1 - 2019/7/31<br>2019/9/1 - 2019/9/30	Annual Mean	VOCs	13.02			[23]
Xingtai(XT)				14.72			
Baoding(BD)				13.74			
Cangzhou(CZ)				7.6			
Chengdu(CD)				21.9			
Handan(HD)				10.27			
Hengshui(HS)				4.79			
Langfang(LF)				5.88			
Shijiazhuang				3.82			
Lanzhou(LZ)	2013/1/1 - 2013/12/31	Annual Mean	VOCs		85.16		[24]
Shenzhen(SZ)	2016/12/16 - 2016/12/31	winter	VOCs		185.3		[25]

Mexico City	2003/4	Spring	VOCs	25			[26]

* Unit of L_{OH} in the table is $cm^3 \cdot molecule^{-1} \cdot s^{-1}$, abbreviated as s^{-1}.Unit of OFP in the table is ppbv O_3. Unit of SOAFP in the table is $g \cdot g^{-1}$.

References

1. Feng, X.; Feng, Y.; Chen, Y.; Cai, J.; Li, Q.; Chen, J. Source apportionment of PM2.5 during haze episodes in Shanghai by the PMF model with PAHs. *Journal of Cleaner Production* **2022**, *330*, doi:10.1016/j.jclepro.2021.129850.
2. Huang, A.; Yin, S.; Yuan, M.; Xu, Y.; Yu, S.; Zhang, D.; Lu, X.; Zhang, R. Characteristics, source analysis and chemical reactivity of ambient VOCs in a heavily polluted city of central China. *Atmospheric Pollution Research* **2022**, *13*, doi:10.1016/j.apr.2022.101390.
3. Magesh, N.S.; Tiwari, A.; Botsa, S.M.; da Lima Leitao, T. Hazardous heavy metals in the pristine lacustrine systems of Antarctica: Insights from PMF model and ERA techniques. *J Hazard Mater* **2021**, *412*, 125263, doi:10.1016/j.jhazmat.2021.125263.
4. Zhang, C.; Liu, X.; Zhang, Y.; Tan, Q.; Feng, M.; Qu, Y.; An, J.; Deng, Y.; Zhai, R.; Wang, Z.; et al. Characteristics, source apportionment and chemical conversions of VOCs based on a comprehensive summer observation experiment in Beijing. *Atmospheric Pollution Research* **2021**, *12*, 230-241, doi:10.1016/j.apr.2020.12.010.
5. Atkinson, R.; Arey, J. Atmospheric degradation of volatile organic compounds. *Chem Rev* **2003**, *103*, 4605-4638, doi:10.1021/cr0206420.
6. Berndt, T.; Böge, O. Gas-phase reaction of OH radicals with phenol. *Phys. Chem. Chem. Phys.* **2003**, *5*, 342-350, doi:10.1039/b208187c.
7. Berg, F.; Novelli, A.; Dubus, R.; Hofzumahaus, A.; Holland, F.; Wahner, A.; Fuchs, H. Temperature-dependent rate coefficients for the reactions of OH radicals with selected alkanes, aromatic compounds, and monoterpenes. *Atmospheric Chemistry and Physics* **2024**, *24*, 13715-13731, doi:10.5194/acp-24-13715-2024.
8. Semadeni, M.; Stocker, D.W.; Kerr, J.A. The temperature dependence of the OH radical reactions with some aromatic compounds under simulated tropospheric conditions. *International Journal of Chemical Kinetics* **2004**, *27*, 287-304, doi:10.1002/kin.550270307.
9. Han, L.; Siekmann, F.; Zetzsch, C. Rate Constants for the Reaction of OH Radicals with Hydrocarbons in a Smog Chamber at Low Atmospheric Temperatures. *Atmosphere* **2018**, *9*, 320, doi:10.3390/atmos9080320.
10. Carter, W. *Updated maximum incremental reactivity scale and hydrocarbon bin reactivities for regulatory applications*; 01/01 2010; pp. 07-339.
11. Venecek, M.A.; Carter, W.P.L.; Kleeman, M.J. Updating the SAPRC Maximum Incremental Reactivity (MIR) scale for the United States from 1988 to 2010. *Journal of the Air & Waste Management Association (1995)* **2018**, *68*, 1301-1316, doi:10.1080/10962247.2018.1498410.
12. Li, J.; Deng, S.; Li, G.; Lu, Z.; Song, H.; Gao, J.; Sun, Z.; Xu, K. VOCs characteristics and their ozone and SOA formation potentials in autumn

and winter at Weinan, China. *Environ Res* **2022**, *203*, 111821, doi:10.1016/j.envres.2021.111821.

13. Li, J.; Deng, S.; Tohti, A.; Li, G.; Yi, X.; Lu, Z.; Liu, J.; Zhang, S. Spatial characteristics of VOCs and their ozone and secondary organic aerosol formation potentials in autumn and winter in the Guanzhong Plain, China. *Environ Res* **2022**, *211*, 113036, doi:10.1016/j.envres.2022.113036.
14. Wang, S.; Zhao, Y.; Han, Y.; Li, R.; Fu, H.; Gao, S.; Duan, Y.; Zhang, L.; Chen, J. Spatiotemporal variation, source and secondary transformation potential of volatile organic compounds (VOCs) during the winter days in Shanghai, China. *Atmospheric Environment* **2022**, *286*, doi:10.1016/j.atmosenv.2022.119203.
15. Wang, G.; Cheng, S.; Wei, W.; Zhou, Y.; Yao, S.; Zhang, H. Characteristics and source apportionment of VOCs in the suburban area of Beijing, China. *Atmospheric Pollution Research* **2016**, *7*, 711-724, doi:10.1016/j.apr.2016.03.006.
16. Hui, L.; Liu, X.; Tan, Q.; Feng, M.; An, J.; Qu, Y.; Zhang, Y.; Jiang, M. Characteristics, source apportionment and contribution of VOCs to ozone formation in Wuhan, Central China. *Atmospheric Environment* **2018**, *192*, 55-71, doi:10.1016/j.atmosenv.2018.08.042.
17. Liu, T.; Hong, Y.; Li, M.; Xu, L.; Chen, J.; Bian, Y.; Yang, C.; Dan, Y.; Zhang, Y.; Xue, L.; et al. Atmospheric oxidation capacity and ozone pollution mechanism in a coastal city of southeastern China: analysis of a typical photochemical episode by an observation-based model. *Atmos. Chem. Phys.* **2022**, *22*, 2173-2190, doi:10.5194/acp-22-2173-2022.
18. Han, S.; Tan, Y.; Gao, Y.; Li, X.; Ho, S.S.H.; Wang, M.; Lee, S.C. Volatile organic compounds at a roadside site in Hong Kong: Characteristics, chemical reactivity, and health risk assessment. *Sci Total Environ* **2023**, *866*, 161370, doi:10.1016/j.scitotenv.2022.161370.
19. Kalbande, R.; Yadav, R.; Maji, S.; Rathore, D.S.; Beig, G. Characteristics of VOCs and their contribution to O3 and SOA formation across seasons over a metropolitan region in India. *Atmospheric Pollution Research* **2022**, *13*, doi:10.1016/j.apr.2022.101515.
20. Yi, X.X.; Li, J.H.; Li, G.H.; Lu, Z.Z.; Sun, Z.G.; Gao, J.; Deng, S.X. [Characteristics of VOCs and Formation Potentials of O(3) and SOA in Autumn and Winter in Tongchuan, China]. *Huan Jing Ke Xue* **2022**, *43*, 140-149, doi:10.13227/j.hjkx.202104180.
21. W.J., Z.R.L.H.W.D.S.X.R.S.J.W. Characteristics of VOCs and formation potential of O3 and SOA in autumn and winter in Baoji,China. *China Environmental Science* **2020**, 983-996, doi:doi.org/10.0000/j.zghjkx.1000-6923.20204016797.
22. Liu, X.; Lu, J.; Li, W.; Liu, Z.; Tong, Y.; Chen, H.; Yu, J.; Ding, Y. Characterization, source apportionment, and assessment of volatile organic compounds in a typical urban area of southern Xinjiang, China.

Air Quality, Atmosphere & Health **2022**, *15*, 785-797, doi:10.1007/s11869-021-01133-4.
23. Guan, Y.; Zhang, Y.; Zhang, Y.; Wang, X.; Han, J.; Song, W.; Hou, L.a.; Duan, E. Pollution Characteristics and Key Reactive Species of Volatile Organic Compounds in Beijing-Tianjin-Hebei Area, China. *Aerosol and Air Quality Research* **2020**, *20*, 1886-1897, doi:10.4209/aaqr.2019.11.0595.
24. Jia, C.; Mao, X.; Huang, T.; Liang, X.; Wang, Y.; Shen, Y.; Jiang, W.; Wang, H.; Bai, Z.; Ma, M.; et al. Non-methane hydrocarbons (NMHCs) and their contribution to ozone formation potential in a petrochemical industrialized city, Northwest China. *Atmospheric Research* **2016**, *169*, 225-236, doi:10.1016/j.atmosres.2015.10.006.
25. Wang, C.; Huang, X.F.; Han, Y.; Zhu, B.; He, L.Y. Sources and Potential Photochemical Roles of Formaldehyde in an Urban Atmosphere in South China. *Journal of Geophysical Research: Atmospheres* **2017**, *122*, doi:10.1002/2017jd027266.
26. Shirley, T.R.; Brune, W.H.; Ren, X.; Mao, J.; Lesher, R.; Cardenas, B.; Volkamer, R.; Molina, L.T.; Molina, M.J.; Lamb, B.; et al. Atmospheric oxidation in the Mexico City Metropolitan Area (MCMA) during April 2003. *Atmos. Chem. Phys.* **2006**, *6*, 2753-2765, doi:10.5194/acp-6-2753-2006.
